# Solvent-Dependent Dynamics of Cellulose Nanocrystals in Process-Relevant Flow Fields

**DOI:** 10.1021/acs.langmuir.4c01846

**Published:** 2024-06-11

**Authors:** Ruifu Wang, HongRui He, Jiajun Tian, Shirish Chodankar, Benjamin S. Hsiao, Tomas Rosén

**Affiliations:** † Department of Chemistry, Stony Brook University, Stony Brook, New York 11794-3400, United States; ‡ National Synchrotron Light Source II, Brookhaven National Laboratory, Upton, New York 11793-5000, United States; § Department of Fiber and Polymer Technology and Wallenberg Wood Science Center, KTH Royal Institute of Technology, SE-100 44 Stockholm, Sweden

## Abstract

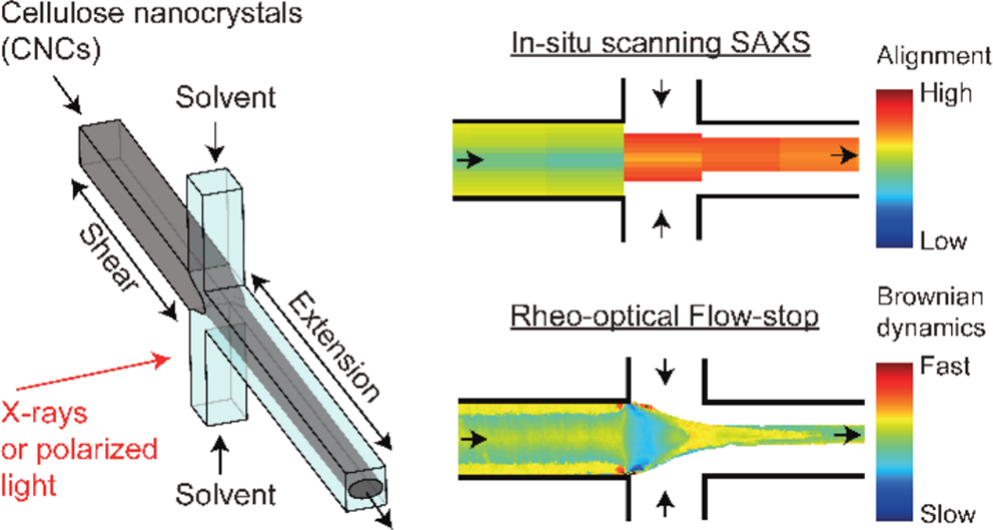

Flow-assisted alignment of anisotropic nanoparticles is a promising route for the bottom-up assembly of advanced materials with tunable properties. While aligning processes could be optimized by controlling factors such as solvent viscosity, flow deformation, and the structure of the particles themselves, it is necessary to understand the relationship between these factors and their effect on the final orientation. In this study, we investigated the flow of surface-charged cellulose nanocrystals (CNCs) with the shape of a rigid rod dispersed in water and propylene glycol (PG) in an isotropic tactoid state. *In situ* scanning small-angle X-ray scattering (SAXS) and rheo-optical flow-stop experiments were used to quantify the dynamics, orientation, and structure of the assigned system at the nanometer scale. The effects of both shear and extensional flow fields were revealed in a single experiment by using a flow-focusing channel geometry, which was used as a model flow for nanomaterial assembly. Due to the higher solvent viscosity, CNCs in PG showed much slower Brownian dynamics than CNCs in water and thus could be aligned at lower deformation rates. Moreover, CNCs in PG also formed a characteristic tactoid structure but with less ordering than CNCs in water owing to weaker electrostatic interactions. The results indicate that CNCs in water stay assembled in the mesoscale structure at moderate deformation rates but are broken up at higher flow rates, enhancing rotary diffusion and leading to lower overall alignment. Albeit being a study of cellulose nanoparticles, the fundamental interplay between imposed flow fields, Brownian motion, and electrostatic interactions likely apply to many other anisotropic colloidal systems.

## Introduction

Alignment and assembly processes for bottom-up fabrication of advanced materials using biobased nanoscale particles as building blocks have the potential to meet the increasing demand of sustainable and environmentally friendly materials. The bulk properties of materials assembled from the anisotropic nanoparticles, such as mechanical and optical properties, are highly dependent on their orientation and microstructures.^1−8^ Understanding and controlling the orientation is thus crucial to tune and enhance the properties of the assembled materials such as filaments,^3,4^ optical films,^5^ hydrogels,^6,7^ and even nanocomposites.^8^ In specific, there is great interest in the use of rod-shaped nanoparticles, which are abundant in nature, ranging from small inorganic particles^9^ to complex large biomacromolecules.^10,11^

**Figure 1:**
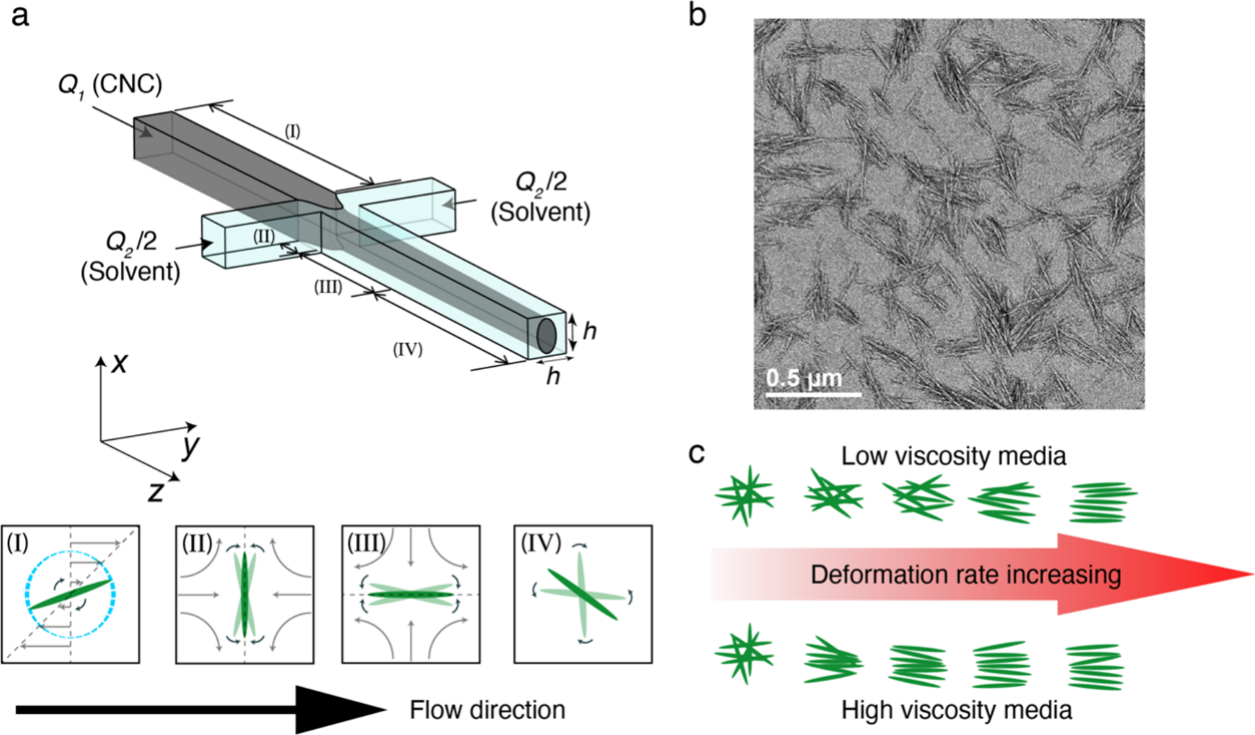
Illustration of the experiment to study dynamics of cellulose nanocrystals (CNCs) during flow-focusing (the flow rate is the same in all of the inlets, i.e., *Q*_1_ = 0.5*Q*_2_ = *Q*). (a) The flow-focusing channel geometry (*h* = 1 mm) and the motion of an anisotropic particle under shear (region I), compressional (region II), extensional (region III), and zero-deformation (region IV) flow fields; the blue dashed circle in (I) represent the tumbling orbit with the thickness reflecting the orientation probability along that direction; (b) the transmission electron microscopy (TEM) image of CNC nanoparticles (adopted from ref (35)); and (c) the influence of viscosity and deformation rate on the alignment of the anisotropic particles.

Although there are several proposed methods of aligning anisotropic colloidal particles, including the usage of electric^12,13^ and magnetic fields,^1,14,15^*hydrodynamic* alignment is a particularly useful and easy method since the flow of the colloidal material can enable continuous material processes. Hydrodynamic alignment of nanoparticles relies on the competition between hydrodynamic torques from velocity gradients (causing rotation and alignment) and the Brownian rotary diffusion of the particles (causing random dealignment). Brownian diffusion is governed by numerous factors, including the sizes/shapes of the particles as well as their interactions both with other particles and solvent molecules.^16,17^ The velocity gradients providing the hydrodynamic torques are generally created by the confined flow of the material process, where different geometries result in different aligning mechanisms at certain positions in the flow.^18^*Shear* flow is the simplest flow field present in any confined pressure-driven flow, with the highest shear rates close to the confining walls. A non-Brownian rod-shaped particle will perform a rotational “tumbling” motion in the shear flow, where the particle spends most of its time in the flow direction but is periodically flipping 180°.^19,20^ Another type of flow field is *extensional* flow, which is present in converging geometries such as nozzles/funnels and will cause an alignment of rod-shaped particles in the extensional direction.^21^ However, since the colloidal dispersion is in contact with the walls, the system will mostly experience shear. In material fabrication processes, shear is undesirable as it will disrupt the nanoscale structure owing to the inherent rotational component.^22^*Flow-focusing* has therefore been proposed as an efficient way to align and assemble nanoparticles in a controlled manner.^23,24^ In this geometry (Figure 1a), the core material is focused by two perpendicular sheath flows, which can cause a detachment from the walls and result in alignment by extensional flow with a low shear. This process is dependent on the rheology of the materials and the flow-rate ratios.

Cellulose nanocrystals (CNCs), negatively charged derivatives of cellulose fibers, constitute an ideal model for the study of the dynamics and orientation of rod-shaped particles during flow. This is because CNCs have small cross-sectional dimensions, large aspect ratio (Figure 1b),^25^ high rigidity, and become birefringent when aligned.^26^ These unique properties make CNCs a good material candidate for oriented optical films with tunable mechanical strength and inks for three-dimensional (3D) printing.^2^ The properties of the CNC-based products are highly correlated with the orientations of CNCs. For example, a high degree of CNC orientation could greatly enhance the tensile modulus in the direction of alignment.^2,27^

There have been numerous studies of CNC microstructures at various concentrations using small-angle X-ray scattering (SAXS)^28^ and polarized optical microscopy^29^ revealing that CNCs can self-assemble into chiral nematic structures at elevated concentrations, both in aqueous and nonaqueous media.^30,31^ Depending on the CNC concentration, a locally ordered mesoscale structure called *tactoid* and glassy cholesteric phase can be formed. The effect of flow gradients on these structures has been studied in a simple shear flow in rheological experiments,^32^ where the structure evolution can also be monitored *in situ*.^33^ For example, using a microfluidic device and *in situ* SAXS, Rosén et al.^23^ studied the orientation of CNCs in a pressure-driven confined flow, which is the typical flow within tubes and pipes. It was found that CNCs behave as Brownian ellipsoids at dilute conditions, but as they assemble into tactoids at higher concentrations, their Brownian dynamics slow down and they become easier to align at lower deformation rates. Calabrese et al.^26^ studied the flow-induced birefringence of dilute isotropic CNCs (0.1 wt %, dispersed in a glycerol/water mixture) under shear and planar extensional fields, finding that dilute CNCs can be aligned much easier in extensional flow than in shear flow. Later, Pignon et al.^34^ used a combination of rheometry and *in situ* SAXS to study structural changes of CNCs at various concentrations upon shearing and cessation of the flow. They observed that mesoscale structures could be effectively disrupted at high shear rates and affect both alignment and rheology. However, even though such studies in ideal flow fields are interesting from a fundamental perspective, a typical flow in a practical material process would be much more complicated with multiple types of deformations and a wide range of deformation rates.

The flow-focusing geometry used in the present work can serve as an important example of a typical material process, which enables the study of the CNC suspensions under varying complex flow fields^23^ at the same time. Here, the dispersion will be first sheared, as shown in Figure 1a region I, where it is then slightly compressed (region II in Figure 1a), followed by a uniaxial extensional flow (region III in Figure 1a) while not being subject to hydrodynamic forcing further downstream (region IV in Figure 1a). In this work, we will limit the study to having the same flow rate in all three inlets, i.e., *Q*_1_ = 0.5*Q*_2_ = *Q*.

In this work, the orientation, structures, and dynamics of CNCs in flow-focusing geometry have been systematically studied. In assembling processes of nanoparticles, it is desired to be at a concentration that allows some mobility of nanoparticles, enabling them to have efficient alignment and flowable dispersions, but still possessing some collective behavior to reduce the influence of Brownian rotary diffusion.^36,37^ For this reason, we choose to study CNCs at a concentration corresponding to the isotropic tactoidal state, where an aligned dispersion becomes dealigned over time. Furthermore, as there has been an increasing interest in dispersing nanocellulose in nonaqueous media,^31,38,39^ we will carefully address the effects on the material process when using a viscous organic solvent, in this case, propylene glycol (PG). The effects on the final alignment during flow-focusing are not trivial. The higher viscosity of PG (η = 44 mPa·s of PG versus η = 0.89 mPa·s of water at room temperature) can cause slower Brownian dynamics (Figure 1c), and the different electrostatic conditions in PG (relative permittivity in PG is ε = 32, while water is ε = 80 at room temperature) would likely affect the tactoid structures as well as the collective Brownian dynamics.

With these targets in mind, scanning-SAXS and rheo-optical flow-stop experiments were used to quantify the alignment, structure, and Brownian rotary diffusion of CNCs during flow-focusing. The knowledge gained from this work not only provides new insights into the processing of nanocellulosic materials but also offers new processing strategies for any bottom-up assembly of anisotropic colloidal building blocks.

## Results and Discussion

The CNCs used in this study were well-characterized by Rosén et al. earlier.^35^ Briefly, the lengths of the CNCs were around 100–200 nm with a mean aspect ratio between 10 and 20. Furthermore, there were no dimensions changing when dispersed in different solvents, which has also been verified in another previous study.^39^ Details of the materials characterization are described in the Materials and Methods section and in Figure S1 of the Supporting Information (SI).

The CNC concentration was carefully selected to be as high as possible while staying isotropic and not transition to a biphasic state.^40^ Additionally, a high CNC concentration can cause the focused flow to transition to a dripping regime.^41^ The selected concentration for CNCs in this study was chosen to be 36 ± 3 mg/mL, similar to our previous work.^35^ This concentration allowed for collective structuring in isotropic tactoids and a stationary focused flow, making the semidilute dispersion ideal for controlled assembly processes.^27,35,36^

### *In Situ* Scanning-SAXS

**Figure 2:**
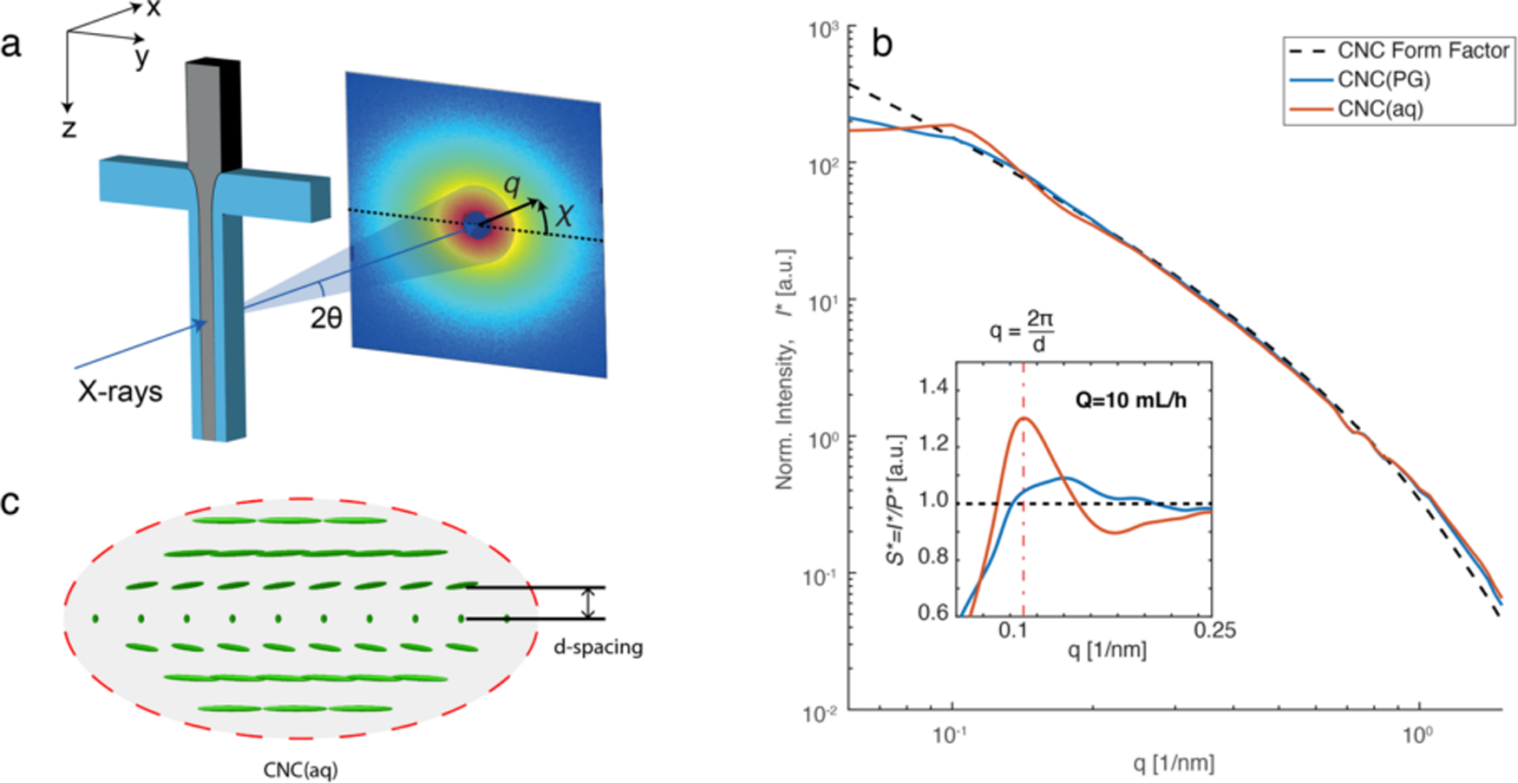
Description of the *in situ* scanning-SAXS experiment. (a) Schematic illustration of the experimental setup; (b) azimuthally averaged and normalized scattering intensity I*(*q*) obtained at the centerline of the flow prior to focusing at *Q* = 10 mL/h of CNC(aq), CNC(PG), and the form factor of dilute aqueous CNC (black dashed line); the inset figure shows the structure factor S*(*q*) of CNC(aq) and CNC(PG) with the peak indicating the distance *d* between the layers of CNCs; and (c) schematic illustration of the chiral nematic tactoidal structure of CNCs.

The *in situ* scanning-SAXS experiments were performed at the 16-ID (LiX) beamline, National Synchrotron Light Source II (NSLS-II), Brookhaven National Lab. A flow cell with the flow-focusing geometry was mounted on a traverse stage, allowing the incoming X-rays in the *x*-direction to be focused on different positions of the channel. The scattered beam intensity *I*(*q*), where *q* = 4π sin θ/λ (2θ being the angle between the incident and scattered beam and λ being the wavelength of the incident beam, see section Materials and Methods), was recorded by X-ray detectors, which could be used to reveal the structure and orientation of CNCs in the flow. An illustration of the experimental setup is shown in Figure 2a.

Figure 2b shows examples of azimuthally averaged and normalized scattering curves I*(*q*) of CNC(aq) and CNC(PG) after background subtraction using a core flow with a pure solvent. The structure factor S*(*q*) in the inset indicates the structural deviation of CNCs from a dilute isotropic system of CNCs (black dashed curve in Figure 2b). The characteristic peak in the structure factor of CNC(aq) indicates tactoidal structures, with the peak position reflecting the lateral spacing *d* = 2π/*q* between layers of CNCs inside the tactoids, as shown in Figure 2c. The structure factor of CNC(PG) suggests that CNCs retain a certain tactoidal structure in PG even with much fewer electrostatic interactions between CNCs and PG, but a broader peak may refer to a less ordered arrangement and generally smaller tactoids.

**Figure 3:**
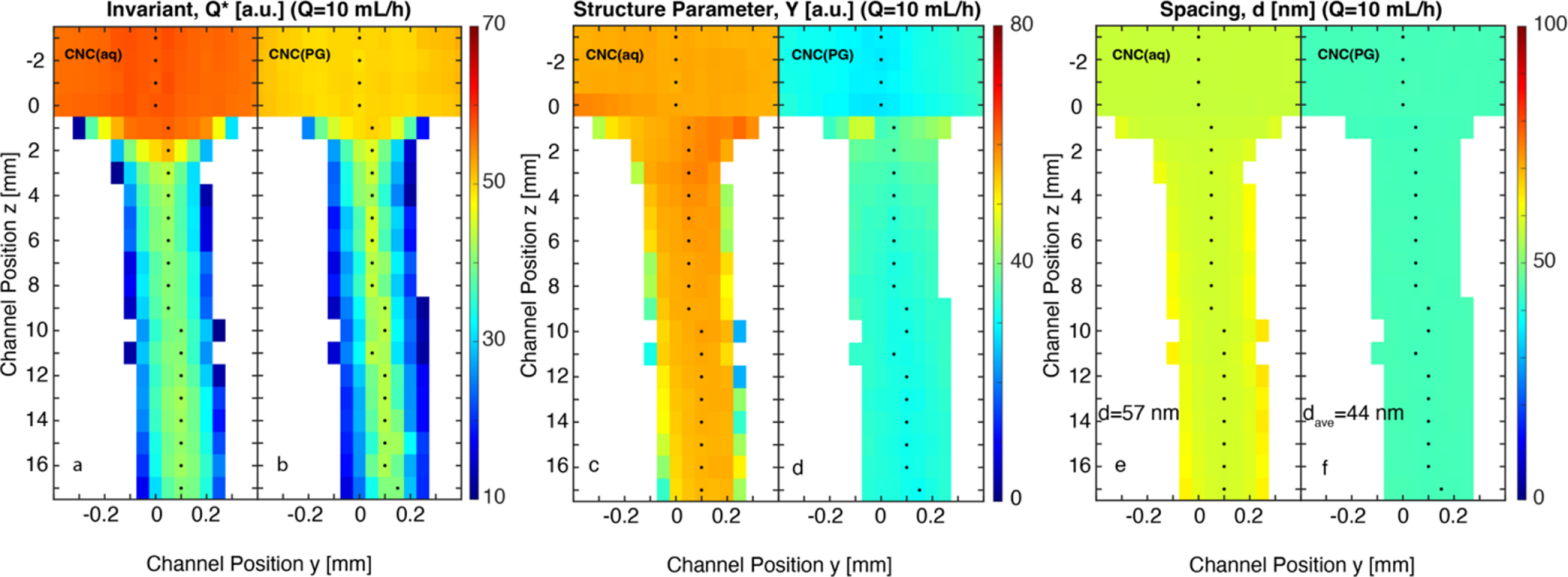
Spatially resolved azimuthally averaged quantities using scanning-SAXS at flow rate *Q* = 10 mL/h for both CNC(aq) and CNC(PG). (a, b) Scattering invariant Q*; (c, d) structure parameter ϒ; and (e, f) spacing *d* between layers of CNCs inside tactoids. Other flow rates are shown in Figures S2–S6.

A scattering invariant, Q* (see the SI for details), was first calculated for each scanning position. Q* represents the scattering power and is a measure of the amount of scattering materials in the volume of the X-ray beam. Figure 3a,b shows two examples of the spatially resolved values of Q* for CNC(aq) and CNC(PG) at *Q* = 10 mL/h, respectively. A centerline is determined for easier comparison of downstream positions using the highest invariant at a certain *z*-position (i.e., the black dots in Figure 3. We note that at *z* < 0, the centerline positions were fixed at *y* = 0.). Despite being at the same concentration, Q* of CNC(aq) was larger than that of CNC(PG) prior to the flow-focusing. This is likely due to the electron density differences between CNCs and the different solvents. The differences of Q* after the focusing region can be attributed to different core ellipticity of CNC(aq) and CNC(PG), respectively, owing to different viscosities and effective interfacial tension between the dispersion and the solvent.^42^

Based on the structure factor S*(*q*) illustrated in Figure 2b, a structure parameter, ϒ, could be calculated to quantify the structural deviation of assembled CNCs from the dilute CNCs (see the SI for details) according to the earlier studies of Rosén et al.^28,43^Figure 3c,d shows the spatially resolved ϒ values of CNC(aq) and CNC(PG) at *Q* = 10 mL/h, respectively. The values of ϒ for both CNC(aq) and CNC(PG) showed a small decrease along the *z*-direction, indicating a slow structural decay toward less organized tactoids. This is likely due to the dilution of the core CNC suspensions by the pure solvent from the sheath flow, as the phenomenon was even more pronounced at the interface between the core and sheath flows. This dilution also caused a slight increase in *d*-spacing near the interface. The spacing *d* (Figure 3e,f) of CNC(PG) was found to be smaller, around *d* = 44 nm, compared to that of CNC(aq) at *d* = 57 nm. Furthermore, both structure parameters ϒ and *d* remained basically constant regardless of the type of deformation (extension/shear) or deformation rates. The spatially resolved quantities of Q*, Y, and *d* at other flow rates are shown in Figures S2–S6.

**Figure 4:**
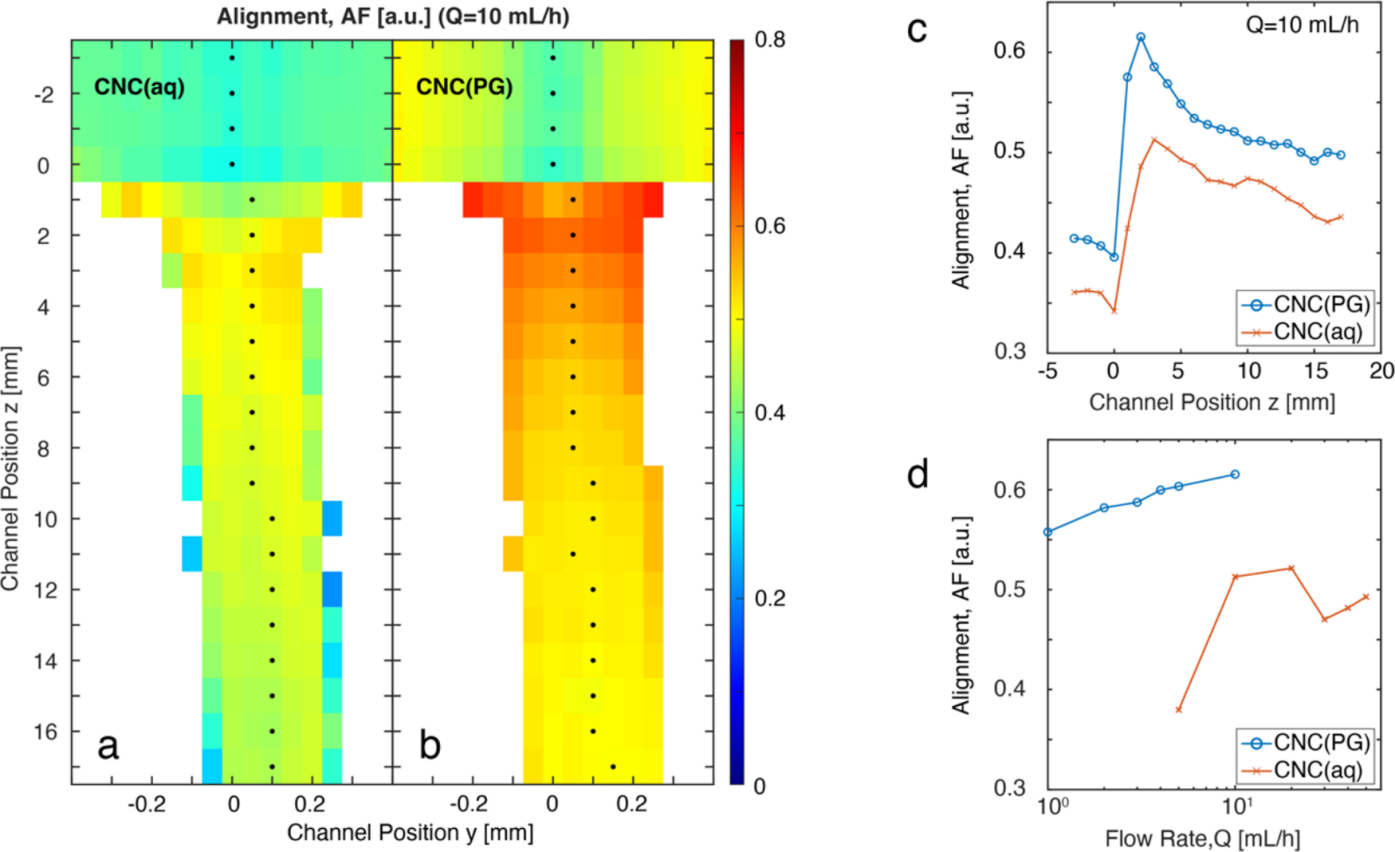
Spatially resolved alignment from scanning-SAXS quantified through the anisotropic factor (AF). (a) CNC(aq) and (b) CNC(PG) at *Q* = 10 mL/h. Other flow rates are shown in Figures S7 and S8. (c) Alignment AF versus downstream position *z* along the centerline of CNC(aq) and CNC(PG) and (d) the maximum centerline alignment AF for CNC(aq) and CNC(PG) at various flow rates.

From the anisotropic scattering pattern, the hydrodynamic alignment of CNCs was quantified using the anisotropic factor (AF) introduced by Pujari et al.^44^ (see the SI for details). This choice allows for measuring the anisotropy without having to specify a reference direction, as opposed to, e.g., the Hermans orientation factor, making it well suited for quantifying alignment of elongated nanoparticles in shear flow, where the orientation distribution is tilted in the gradient direction.^35^ The values of AF are illustrated in Figure 4a,b at *Q* = 10 mL/h. Other flow rates are shown in Figures S7 and S8. Figure 4c shows the alignment along the centerline of CNC(aq) and CNC(PG), respectively, which can be used to highlight the different regions described in Figure 1. In region I, before focusing, the system is aligned by shear, with a higher alignment close to the walls due to higher shear rates. The drop in AF at *z* = 0 reflects region II, where the compression causes a rapid dealignment. The strong alignment due to extensional flow in region III becomes visible shortly thereafter, followed by decay due to Brownian rotary diffusion in region IV when a plug flow is formed at the center with no velocity gradients. There is an interesting difference in terms of alignment between the CNC(aq) and CNC(PG) systems, as illustrated with the maximum centerline AF in Figure 4d. Owing to the higher viscosity of the solvent, it is clear that the CNC(PG) can reach much higher degrees of alignment at very low flow rates. Even at *Q* = 1 mL/h, the maximum alignment of AF = 0.56 is higher than the highest alignment in the CNC(aq) system of AF = 0.53 at *Q* = 20 mL/h. While the maximum AF of CNC(PG) monotonically increased with flow rate, CNC(aq) showed a more counterintuitive nonmonotonic trend. The maximum AF of CNC(aq) decreased when the flow rate increased from *Q* = 20–30 mL/h and then started increasing again at higher flow rates but was still lower at *Q* = 50 mL/h than maximum AF at *Q* = 20 mL/h. To examine the underlying dynamics of this behavior in detail, other characterization techniques were used.

### Rheo-Optical Flow-Stop

**Figure 5:**
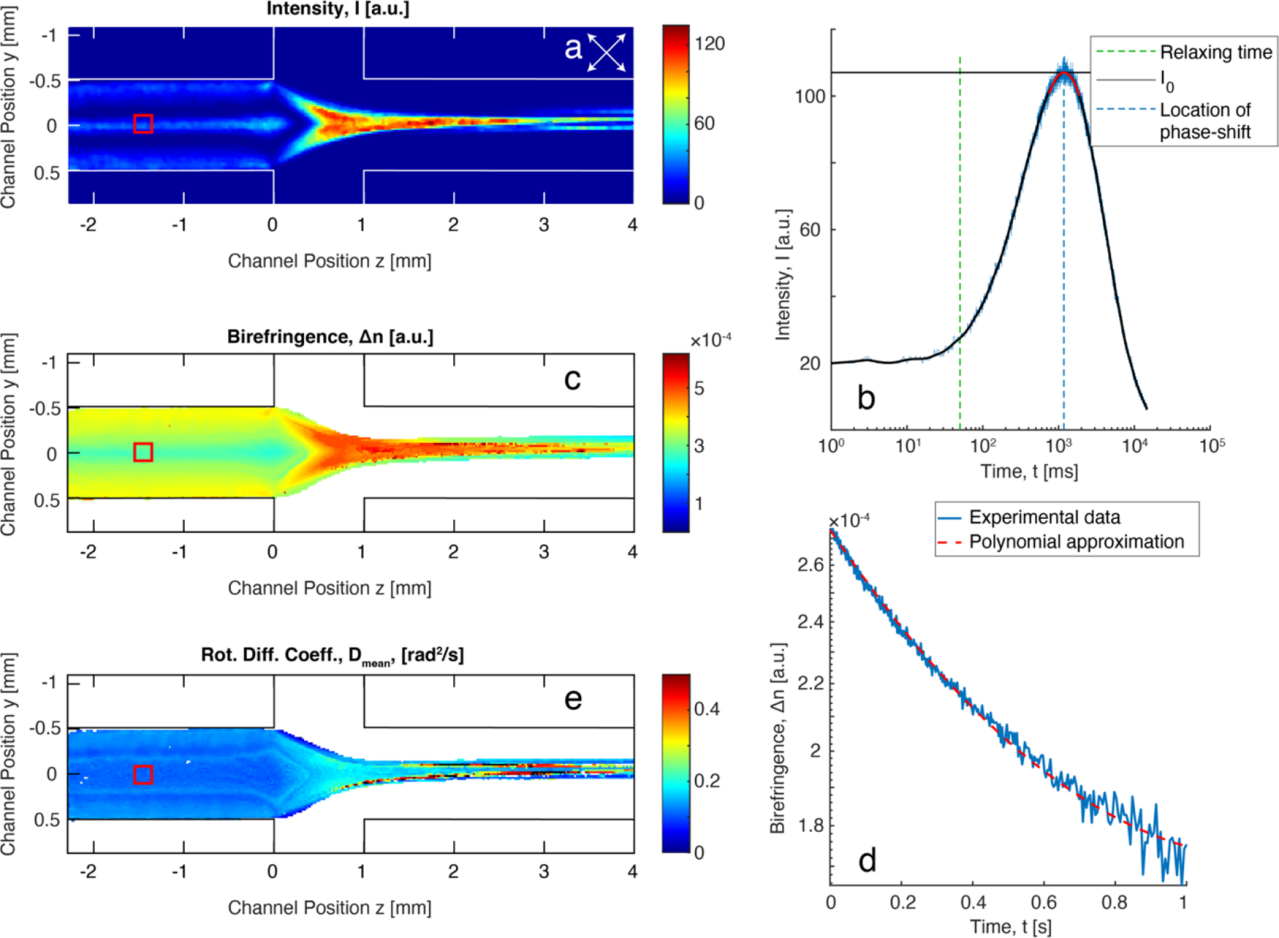
Illustration of the rheo-optical flow-stop experiment of CNC(PG) at *Q* = 10 mL/h. (a) Spatially resolved values of intensity during flow; (b) the intensity evolution in the red square in (a) after stopping the flow; (c) spatially resolved values of Δ*n* (higher Δ*n* corresponds to higher CNC alignment); (d) the decay of Δ*n* after stop in the red square and the polynomial fit according to the cumulant expansion; and (e) spatially resolved values of *D*_mean_ (higher values means that CNCs have faster Brownian rotary motion).

While scanning-SAXS is a powerful tool to characterize the orientation and structure of flowing CNCs, it lacks the ability to reveal the time-resolved Brownian motion of CNCs in the confined flow. Therefore, we studied the same materials using a rheo-optical flow-stop device to reveal the relationship between the dynamics and the structure of CNCs through their Brownian rotary diffusion.^23,28^ In brief, the same flow cell as used for scanning-SAXS was placed between two cross-polarized linear polarizers having −45 and +45° of polarizing angle with respect to the flow direction (the flow direction is the same as the gravity direction; see Figure S9). A red laser (λ ≈ 660 nm) was used as a light source, and 4 three-way valves were connected to the flow cell inlets/outlet to stop the flow and trigger the relaxation procedure of CNCs from aligned to isotropic state (see section Materials and Methods and SI for details). When the system of CNCs is aligned, it becomes birefringent, and the birefringence Δ*n* can be quantified through the light intensity:
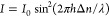
1passing through the system. Figure 5a shows the intensity distribution recorded by a high-speed monochromatic camera of CNC(PG) during a stationary flow at *Q* = 10 mL/h. As the CNCs relax toward isotropy after stopping the flow, the birefringence decays exponentially according to Δ*n*(*t*) ∝ exp(−6*D*_r_*t*), with *D*_r_ being the rotary diffusion coefficient. The strong birefringence of CNCs causes phase shifts of intensity according to eq 1, which are visible as an oscillating signal during the decay (see Figure 5b). This can be used to find *I*_0_ through the location of the phase shifts, and the results can be converted into quantitative values of Δ*n*, as seen in Figure 5c (more details in SI and Figure S10). The decay of birefringence Δ*n*(*t*) illustrated in Figure 5d shows that the dynamic behavior is not fully exponential owing to different particle sizes and collective structures in the system, causing a polydispersity of dynamics. To analyze these data, we use a second-order cumulant expansion to obtain a mean rotary diffusion coefficient *D*_mean_ and polydispersity index (PDI; see the SI for details and Figure S11 for PDI) for the different pixels in the image. The resulting spatially resolved values of *D*_mean_ are illustrated in Figure 5e. It is noted that there are some artifacts remaining in the regions close to an initial phase shift where *I* ≈ 0 and the initial decay of Δ*n*(*t*) is not easily obtained to estimate the corresponding *D*_mean_.

**Figure 6:**
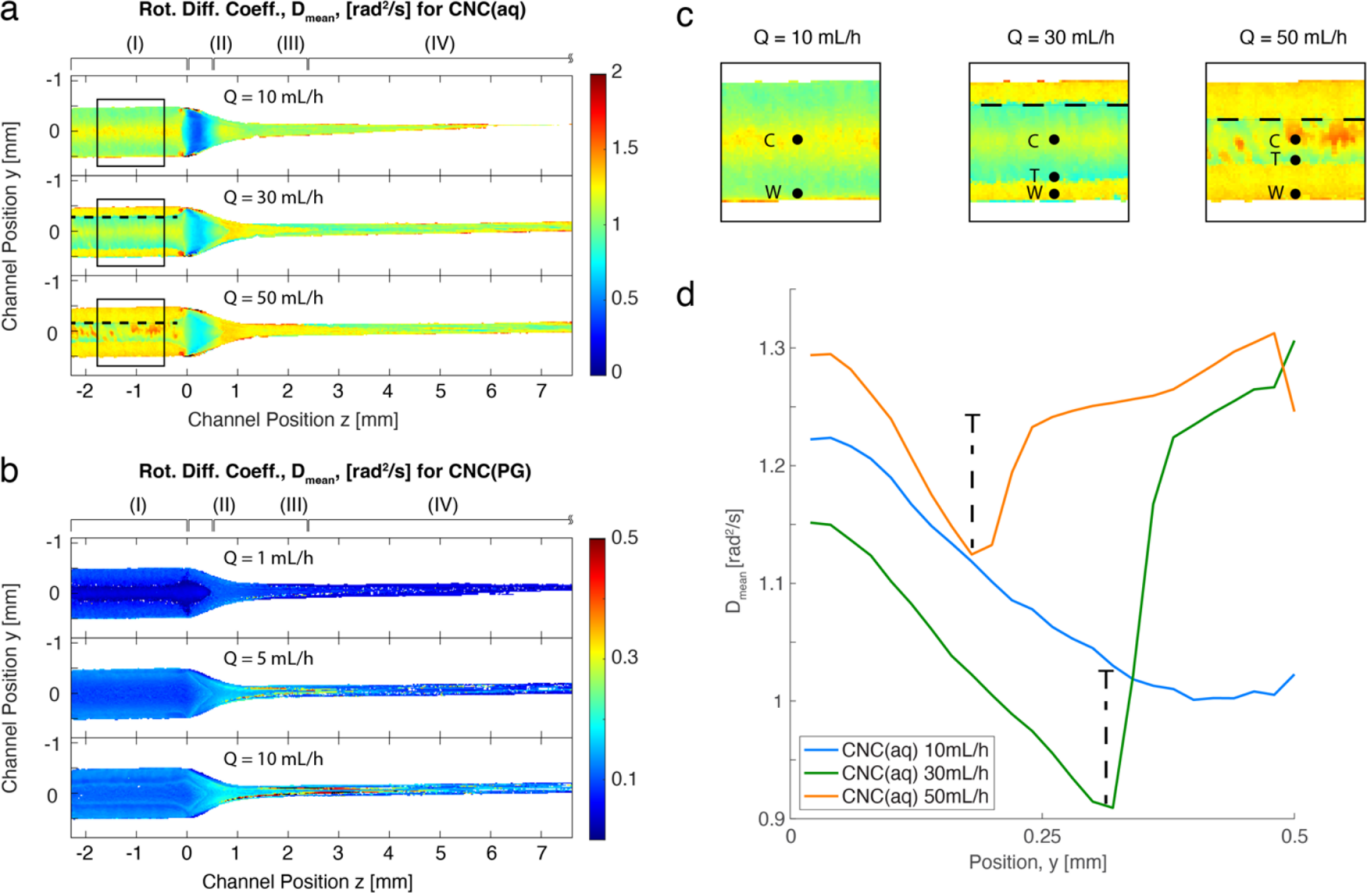
Results from the rheo-optical flow-stop experiment. (a) Spatially resolved values of *D*_mean_ for CNC(aq) at *Q* = 10, 30, and 50 mL/h, respectively; the dashed lines highlight the boundary of the sheared region; (b) spatially resolved values of *D*_mean_ of CNC(PG) at = 1, 5, and 10 mL/h, respectively; (c) zoom-in on region I for CNC(aq) with positions for center (C), wall (W), and transition (T) denoted in figures; and (d) the *z*-direction-averaged *D*_mean_ of CNC(aq) in region I as a function of the *y*-position.

Figure 6a,b shows the *D*_mean_ values for CNC(aq) and CNC(PG) at various flow rates, respectively, which elucidate the collective dynamics of CNCs in the system. Since there is some apparent polydispersity in the system, it is natural to assume that higher values of *D*_mean_ are present in regions of higher deformation (shear/extension) rates,^35^ i.e., close to the walls in region I and in the extensional flow of region III, and also at higher flow rates. This is because the higher hydrodynamic forces can align smaller objects with stronger Brownian motion. This behavior can also be seen in the CNC(PG) system in Figure 6b. However, CNC(aq) at *Q* = 10 mL/h showed the opposite trend in region I, with lower *D*_mean_ close to the walls compared to the centerline. This phenomenon will be discussed later. In region II, there was a sudden drop in *D*_mean_ caused by compression, which could be linked to the lower AF values in the same region (see Figure 4). In regions III and IV, the *D*_mean_ value of CNCs exhibited the expected behavior: *D*_mean_ first increased due to the stronger extensional flow, followed by a decrease due to the stronger Brownian motion of smaller aligned objects in region III.

For higher flow rates, a transition is clearly observed in region I for CNC(aq), as illustrated in the enlarged figures in Figure 6c. At *Q* = 30 and 50 mL/h, we can observe a layer of higher *D*_mean_ at around 1.3 rad^2^/s close to the walls of region I, which we term the “sheared region” for CNC(aq) with the boundary drawn with a dashed line in the figure. Interestingly, it appears that only the thickness changed with the flow rate but not the value of *D*_mean_ inside this layer. Another interesting observation is the patches of fast dynamics in the center part of region I at *Q* = 50 mL/h, which reflect the inhomogeneous structure of the sheared regions at the windows observed in the gradient direction. To illustrate this dynamic behavior in region I, the *z*-averaged value of *D*_mean_ is shown in Figure 6d, where there is a clear transition point (*T*) from decreasing to increasing values of *D*_mean_ with the distance from the center (C) to the wall (W).

**Figure 7:**
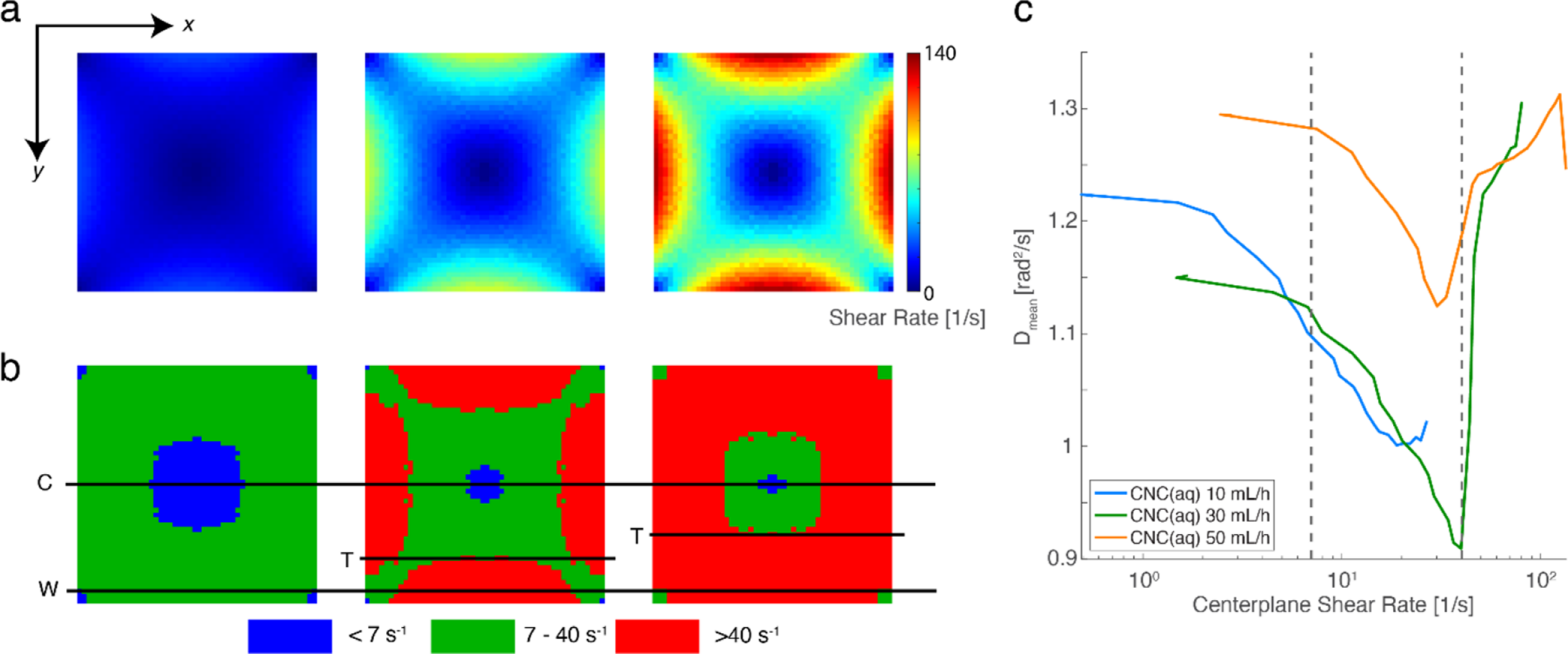
Results from CFD simulations. (a) Simulated shear rate distribution in the channel cross-section at *Q* = 10, 30, and 50 mL/h; (b) classification of three regions based on simulated shear rate, with the same positions noted as in Figure 6c; and (c) the *z*-direction-average *D*_mean_ of CNC(aq) in region I as a function of centerplane shear rate.

To understand this further, we used computational fluid dynamics (CFD, details in SI and Figure S12) to estimate the shear rates in region I and link to the observed diffusive behavior, with results illustrated in Figure 7a. Based on the observed dynamics, we classified three regions by their shear rates in Figure 7b: low shear rate (blue) <7 s^–1^, moderate shear rate (green) 7–40 s^–1^, and high shear rate (red) > 40 s^–1^. At *Q* = 10 mL/h, at the center position (C), the observed dynamics would be mostly from the low shear region, where we also see relatively high values of *D*_mean_, which we can assume are close to the actual rotational diffusion of the system at rest. Moving from the center position (C) to the wall position (W), the system experiences higher shear rates, and there is a corresponding decreasing trend of *D*_mean_. We can thus assume that the region of moderate shear rates includes physical mechanisms where *D*_mean_ is a function of the shear rate. At *Q* = 30 mL/h, the observed dynamics at the wall position is dominated by the high shear rate region, which is linked to the rapid increase of *D*_mean_. From Figure 7b at *Q* = 30 mL/h, it is clear that there is likely an expected transition (*T*) when the centerplane shear rate has reached the critical value, which is then closer to the center at *Q* = 50 mL/h. Plotting the *D*_mean_ data from Figure 6d as a function of centerplane shear rate, we find that the transition point of both *Q* = 30 and 50 mL/h line up at a critical value around 40 s^–1^. Moreover, the highest *D*_mean_ ≈ 1.3 rad^2^/s at the sheared region, as shown in Figure 7c, seems to be rather flow-rate independent (see specifically Figure S13). It is also important to note that the centerplane shear rate is not a good measure of the average dynamics at the center (C) position, as it is affected by the higher shear rate regions present through the line of sight (see Figure 7b).

### Solvent-Dependent Dynamics under Flow

**Figure 8:**
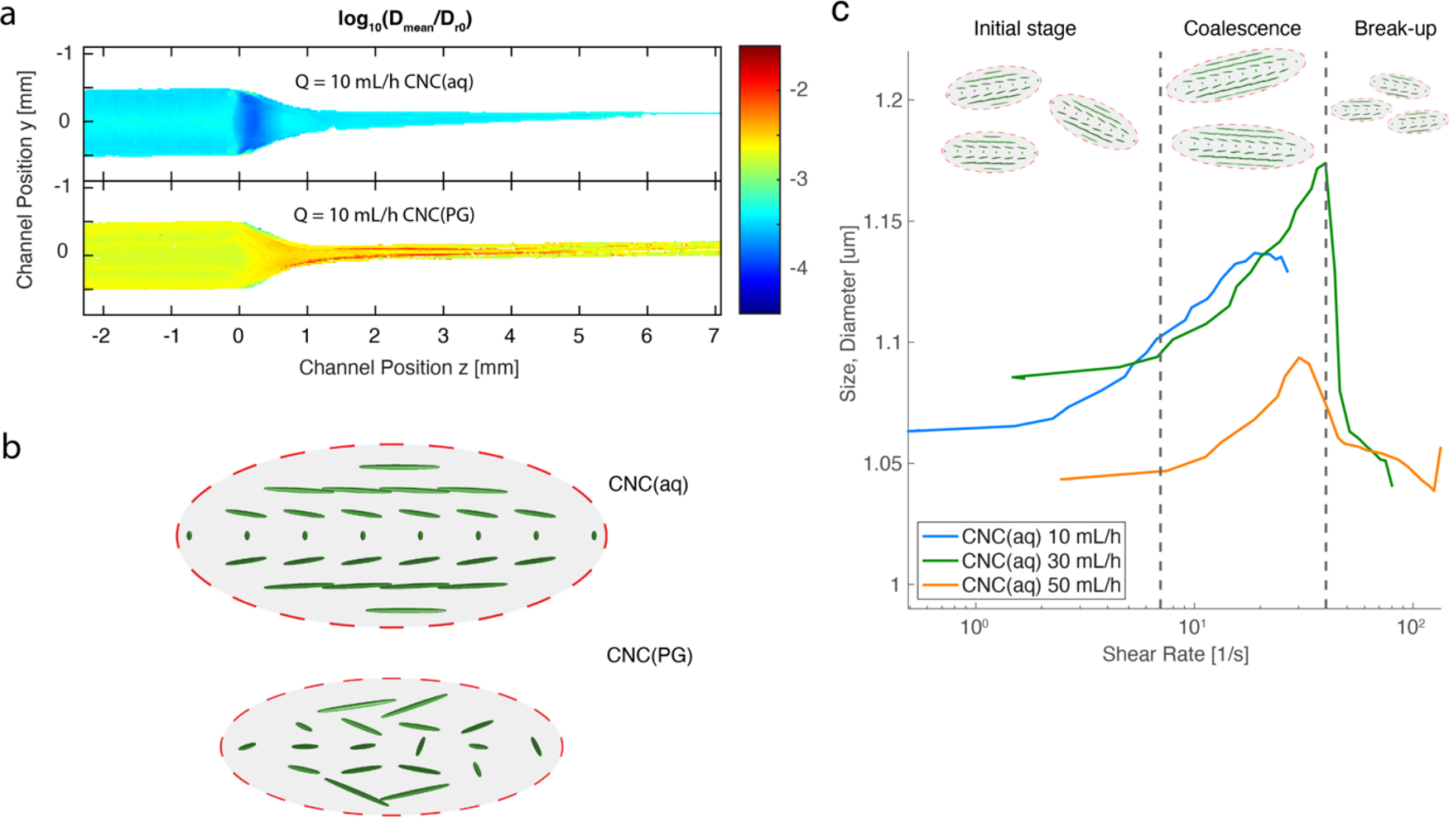
Illustration of the differences in collective dynamics between CNC(aq) and CNC(PG). (a) The ratio of *D*_mean_/*D*_r,0_ plotted logarithmically at *Q* = 10 mL/h; (b) schematic illustration of the differences in ordering between tactoids in water and PG; and (c) apparent tactoid size as a function of centerplane shear rate.

The results presented here illustrate the intricate interplay between the imposed flow fields and the collective ordering of the dispersed CNCs in a typical material process, consisting of regions of shear, extensional, and compressional flow. The CNC(PG) system largely follows the expected behavior of a polydisperse system of Brownian rods, displaying higher values of AF and *D*_mean_ in regions of stronger shear and extension rates. Although a constantly increasing trend of maximum AF was observed in Figure 4d with respect to the flow rate, it cannot reach perfect alignment, as this is limited by the total extension of the system during focusing with the upper limit. The same also applies to the shear flow, where the upper limit of alignment is set by the rate-independent Jeffery-orbits,^45^ which only depend on particle morphology. Similarly, the maximum value of *D*_mean_ is limited by the *D*_r,0_ expected from a system of dilute particles. In the case of CNC(PG), this value is around *D*_r,0_ ≈ 50 rad^2^/s, which is more than 100 times larger than the measured values in the rheo-optical experiment (see the SI for details of the calculation). Hence, there are clearly interactions involved in the system that slow down the Brownian motion. However, a similar estimation for the CNC(aq) system reveals a factor 1000 times slower dynamics compared to the theoretical estimation in dilute conditions. This difference is illustrated more clearly in Figure 8a, where values of *D*_mean_/*D*_r,0_ are calculated throughout the channel in both systems at *Q* = 10 mL/h. Considering the results from the SAXS experiments, we can already establish that the collective ordering is much stronger in water than in PG (illustrated in Figure 8b), which likely is the underlying mechanism behind the observed differences in dynamic behavior.

The major difference between CNCs in PG compared to those in water is that the less dissociated charges in the former generally result in smaller tactoids, smaller *d*-spacing, and less collective ordering. However, even a small amount of dissociated charges can still prevent the aggregation of CNCs in PG. This was also seen in similar systems studied by Isogai et al.^46^ and Dorris et al.,^38^ where they pointed out that systems of nanocellulosic particles still possess electrostatic dissociations even in nonaqueous solvents such as dimethylformamide. In a related study, Attia et al.^31^ reported on the structure of CNCs in ethylene glycol (EG), which resulted in smaller collective structures than CNCs in water. However, the *d* values in their reports increased with increasing EG content, which is the opposite of the trend that we observe here. A vicinal water layer and concentration fluctuation of the local water layer were assigned as the reasons for the *d* increment. Here, in CNC(PG), a trace amount of water can be even closer to the surface of CNCs because of similarities in polarity and hydrophilicity, and high viscosity could also suppress the fluctuation of the local water concentration. Hence, the van der Waals interactions become strong enough to hold a *d* value smaller than that of CNCs in water. Establishing this difference on the particle level, we can now turn our attention to the collective ordering in tactoids.

There are a few previous works that can aid us in understanding tactoids in our system. Pignon et al.^34^ demonstrated the breakup and deformation of CNC tactoids under simple shear flow, while Almohammadi et al.^47^ tuned the volume of amyloid fibril tactoids using extensional flow. Both works pointed out that the tactoids can deform and break up if subjected to external deformation. Furthermore, Wang et al.^11^ observed the coalescence of CNC tactoids, where smaller tactoids fuse into larger ones, similar to the coalescence of oil droplets in emulsions. Moreover, this coalescence was more often seen in the polymer blend.^48^ Inspired by these reports, we propose a scenario to explain the behavior in our system of aqueous CNCs.

As the CNCs form tactoids, the rotary diffusion coefficient decreases as the collective motion makes the CNCs act like bigger objects. A rough assumption would be that the collective rotary diffusion coefficient would be similar to that of spherical particles according to the Einstein–Smoluchowski relation:
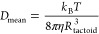
with temperature *T* = 297 K and viscosity η = 8.9 × 10^–4^ Pa·s. Using this relation, we can instead interpret our *D*_mean_ data in Figure 8c in terms of apparent tactoid sizes.

At lower flow rates (*Q* = 10 mL/h), in region I, the system is subjected to only shear flow with stronger shear rates close to the walls. The shear flow causes relative velocities of nearby tactoids, which can come close to each other. At low shear rates, these domains can pass each other without interaction, but at higher rates of inertia, they can collide and coalesce into larger tactoids. This would explain the slower rotary diffusion closer to the walls and the regime of decaying *D*_mean_ with the increasing shear rate seen in Figure 8c. The coalescence mechanism of intermediate-sized tactoids competes with the shear-induced breakup mechanism reported by Pignon et al.,^34^ and at a certain critical value of shear rate, the breaking up of tactoids dominates. At this point, the *D*_mean_ increases rapidly and explains the formation of the “sheared layers” at higher flow rates in Figure 6a. According to Wang et al.,^11^ the tactoids could possibly also merge and form ordered layers along the walls. As these layers break up due to strong shear rates, it is likely that an inhomogeneous structure of fast- and slow-moving patches emerges, as visible in Figure 6a. The probability of breaking a tactoid at a certain shear rate is likely proportional to the tactoid size. The sizes of the broken-up tactoids are likely to be sufficiently small that they become stable with the variation of shear rate in our experiment, which could explain the apparent upper limit to *D*_mean_.

As the sheared dispersion enters region II with compressional flow, the tactoids have an even stronger tendency to coalesce, which is visible in Figure 6a, with a dramatic decrease in *D*_mean_ as the system is compressed. As the compressed dispersion gets rapidly extended in region III by the sheath flows, the tactoids break up again and typically reach the same sizes as in the sheared layers. The emergence of sheared layers and breakup of tactoids in region I thus naturally limit the ability to align the tactoids in region III, which is the ultimate reason for the drop of AF visible in Figure 4d at higher flow rates. Although having a dramatic influence on the alignment in the process, the breakup of tactoids is not easily observed with other techniques. Complementary steady shear rheology revealed a slightly stronger shear-thinning tendency above a critical shear rate of around 50 s^–1^ (see Figure S14), which is consistent with the estimated critical shear rates in the rheo-optical experiment and can be attributed to the same effect. Still, despite these dramatic morphological structural transitions on a mesoscopic scale, it is rather invisible on a nanometer scale as probed with scanning-SAXS. This further underscores the importance of combining dynamic rheo-optical microscopic measurements with nanoscale characterization to fully understand and control the material process on all scales.

## Conclusions

One of the most effective strategies to align and assemble dispersed rodlike nanocellulose particles in continuous processes is through flow-focusing, where a core flow of a semidilute dispersion is accelerated by two perpendicular sheath flows. The assembly can be triggered by the addition of metal ions or acids in the sheath flows, leading to a fixation of the flow-induced structures. In this work, we used scanning-SAXS combined with rheo-optical flow-stop to elucidate the complex interplay of orientation, Brownian motion, and collective ordering *in situ* in such a process for dispersed CNCs both in water and PG. In an aqueous system, CNCs organize in tactoids, which reduces Brownian motion and makes them easier to align at lower flow rates. However, it was found that the maximum alignment that could be achieved is severely limited by the shear-induced breakup of tactoids prior to focusing. Our results suggest that there is a suitable range of shear rates that can promote tactoid coalescence and enable the mesoscopic alignment of CNCs in the subsequent extensional flow by using compressional forces. The CNC alignment in this process could be further improved by using a more viscous solvent such as PG, which can decrease the Brownian motion. However, due to weaker electrostatic interactions and weaker collective tactoid formation in CNC(PG), there is no further improvement from the collective mechanisms found in CNC(aq). The improvement of alignment must also be critically evaluated with respect to the increasing energy demand when a more viscous solvent is used.

In conclusion, it is likely that an arbitrary continuous material process of aligning and assembling nanoscale particles in flowing systems poses its own challenges depending on the system, where the type of collective ordering depends on factors different from the ones presented here. Still, we believe that our work can serve as a guide into how *in situ* measurements on both nano- and macroscale in process-relevant flow situations can be used to capture all of the complexity involved in the process. Finally, we also believe that the strategy of combining rheo-optical flow-stop experiments and scanning-SAXS will not be limited to material processes but can also be applied to other complex flow-induced phenomena, including various polyelectrolytes with complex structures such as DNA, polyoxometalates, and proteins and gain new knowledge.

## Materials and Methods

### Samples

Cellulose nanocrystal (CNC) stock suspension at a concentration of around 11 wt % or 110 mg/mL was used in this study. These samples were obtained from the Process Development Center, University of Maine. 1,2-propaendiol (propylene glycol, PG) was obtained from VWR Chemicals. All chemicals were used without further purification. Deionized (DI) water was used throughout the project to dilute the aqueous suspensions. In the current study, the stock CNC suspension was prepared at 36 mg/mL, corresponding to an isotropic tactoidal state in an aqueous environment.^36^

### Solvent Exchange

The solvent exchange process was carried out using the procedures described in our previous study.^39^ Briefly, PG was mixed with the stock CNC suspension in a 1:1 volume ratio to ensure the same concentration of the CNC(PG) suspension (36 mg/mL). The mixture was then gently heated at 60 °C for ∼12 h to remove the water content, where the mass loss (due to water evaporation) was monitored by an analytical balance. The evaporation of PG was neglected, considering the boiling point of PG (188 °C).^39^

### Flow Cell

The flow cell in this study is identical to the one used by Rosén et al. in another study.^35^ In this setup, the center channel plate was sandwiched between two layers of transparent cyclic olefin copolymer (COC) films (Tekni-Plex 8007 X-04, 150 μm thickness). The low SAXS scattering and low birefringence of the COC films made it perfect for both *in situ* scanning-SAXS and rheo-optical experiments. Furthermore, two 10 mm aluminum plates were screwed and clamped together for mechanical support of the flow cell. The flow-focusing channel geometry consists of a four-channel crossing, each having 1 × 1 mm^2^ in cross-sectional dimensions where three serve as inlets and one outlet. The liquid samples were pumped by NE-4000 programmable syringe pumps. In the focusing region, the CNCs are accelerated by their corresponding solvents (water for CNC(aq) and pure PG for CNC(PG)) injected from the side channels. The core flow rates (*Q*_1_) for CNC(aq) suspensions were 5, 10, 20, 30, 40, and 50 mL/h, and flow rates for CNC(PG) suspensions were 1, 2, 3, 4, 5, and 10 mL/h. The sheath flow rates were set the same as the core *Q*_1_ = 0.5*Q*_2_ = *Q*, leading to a total flow rate of 3*Q*.

### *In Situ* SAXS Experiments

*In situ* SAXS measurements of the flow-focusing geometry during stationary flow were conducted to study the orientation distribution and nanoscale structures of CNCs. The experiments were conducted at the 16-ID beamline at National Synchrotron Light Source II (NSLS-II), Brookhaven National Lab. A traversing stage was used to mount the flow cell, allowing for the scanning-SAXS at different positions of the flow cell. The wavelength of the selected X-ray was λ = 0.9 Å, and the sample-to-detector was 3.6 m, thus covering the *q* range of 0.06–2 nm^–1^, where *q* = 4π sin(θ)/λ (2θ being the angle between incident and scattering beam). The beam size was approximately 50 × 50 μm^2^, and the scattered X-ray was detected by a Pilatus 1 M detector, which has a pixel size of 172 × 172 μm^2^. The flow direction was denoted as the *z*-direction, which is aligned with the direction of gravity, where the incident direction of the X-ray is defined as the *x*-direction. As a comparison, the form factor *P*_0_(*q*) of dilute aqueous CNCs (4 mg/mL, or 0.4 wt %) in an isotropic state was adopted from Rosén et al.^35^ The exposure time was 1 s for each position with 21 at the *z*-direction and 17 at the *y*-direction (total 357 points) covering both upstream (before flow focusing) and downstream (after flow focusing). The scanning-SAXS experiment took approximately 20 min. For the background subtraction, the suspensions in the core flow were simply replaced by the pure corresponding solvents. The background intensity was scaled by a factor (close to 1, according to the transmitted beam intensity) prior to subtraction. All of the experiments were conducted at room temperature (∼293 K).

The CNC samples used in this study were well-characterized earlier by Rosén et al.^35^ using TEM. Briefly, the lengths were typically in the range of 100–200 nm, with the aspect ratios ranging between 10 and 20. SAXS experiments were also conducted in this study for static CNC(aq) and CNC(PG) samples at 5 mg/mL to reveal the dimensional information on their cross sections. The CNCs in water were measured, where the mean cross-sectional dimensions were 30.7 × 3.5 nm^2^ by fitting with a polydisperse parallelepiped model. We note that these results closely match the results in our previous work.^35^ Similarly, CNCs in PG had mean cross-sectional dimensions of 37.0 × 3.1 nm^2^. The tiny dimensional deviations of CNC in PG from CNC in water could be due to the hydration layer on the surface of CNC, as the solvent-exchanging process could not remove the water completely. However, these dimensional changes are considered very negligible, where the assumption that the cross-sectional dimensions of CNCs remain unchanged in two solvents (PG and water) has been verified in accordance with our previous results.^35^ Moreover, as the aim of this research is to discuss the differences between CNC(aq) and CNC(PG) from the ideal structure of CNC triggered by different solvents, the form factor of dilute CNC(aq) was used for the following discussion. The concentration of CNC(PG) in this section was confirmed to be 36 ± 3 mg/mL by gravimetric measurement. The details of the analysis of the SAXS data and the distribution of cross-sectional dimensions are shown in SI and Figure S1.

### Rheo-Optical Flow-Stop Experiments

The flow-stop experiment to extract the rotary diffusion coefficients from hydrodynamically aligned nanomaterials was described by Rosén et al.,^23^ and the instrument is illustrated in Figure S9 (in SI). The flow cell was placed between two linear polarizing films, with polarizing directions +45 and −45 to the *z*-direction (flow direction), respectively. A red laser module was used as the light source (660 nm, 130 mW, 14 mm of spot size) along the *x*-direction (light path direction), and the final intensity was recorded by a high-speed monochrome camera (Mako U-029b). The image acquisition was set at 1500 frames per second for CNC(aq) in 36 mg/mL for 15 s and 1000 fps for CNC(PG) in 36 mg/mL for 25 s. Four synchronized three-way valves (Takasago MTV-3SL) were used to stop the flow instantly after 5 s of stable flow. Videos of the experiments are provided as the Supporting Information.

The intensity *I* is related to the birefringence of the material Δ*n* through the relation in eq 1. Furthermore, the birefringence Δ*n* is proportional to the alignment of the CNCs, i.e., *S*_ϕ_ ∝ Δ*n*, where *S*_ϕ_ is the Hermans orientation factor,  (ϕ being the angle between the direction of the CNC major axis and the flow direction). After the stop of the flow, the birefringence decays due to Brownian rotary diffusion, and thus, the rotary diffusion coefficient can be obtained using the intensity decay in single pixels (details and figures are provided in the SI). Matlab 2019b was used to extract the rotary diffusion coefficient and polydispersity index (PDI) through a second-order polynomial fitting of the intensity decay curve using the method of cumulants. The initial 1000 ms after the stop was used to evaluate the mean rotary diffusion coefficient and polydispersity index. Details of the experiments and analysis are found in the SI.

## Supporting Information Available

Strong birefringence of the material (AVI)
Static SAXS experiments; post-processing of scanning-SAXS experiment; spatially resolved quantities from scanning-SAXS at other flow rates; rheo-optical flow-stop; theoretical estimation of *D*_*r*,0_; computational fluid dynamics (CFD) (PDF)

## Supplementary Material

la4c01846_si_001.avi
