# Supplementary material for: Solvent-Dependent Dynamics of Cellulose Nanocrystals in Process-Relevant Flow Fields
Source: Langmuir. 2024 Jun 11;40(25):13319–29. doi: 10.1021/acs.langmuir.4c01846 (PMC11210288; doi:10.1021/acs.langmuir.4c01846)
Supplement: Supplementary file 2 — la4c01846_si_002.pdf [file la4c01846_si_002.pdf]

Supporting information for:

Solvent-Dependent Dynamics of Cellulose Nanocrystals in Process-Relevant Flow Fields

Ruifu Wang,[§] HongRui He,[§] Jiajun Tian,[§] Shirish Chodankar,[†] Benjamin S. Hsiao[§,*], and Tomas Rosén,[¶,*]

[§]Department of Chemistry, Stony Brook University, Stony Brook, New York, 11794-3400, United States

[†]National Synchrotron Light Source II, Brookhaven National Laboratory, Upton, New York 11793-5000, United States

[¶]Department of Fiber and Polymer Technology and Wallenberg Wood Science Center, KTH Royal Institute of Technology, SE-100 44 Stockholm, Sweden

Static SAXS Experiments

The small-angle X-ray scattering (SAXS) experiment for static CNC sample was conducted at LiX beamline (16-ID), Nation Synchrotron Light Source II (NSLS-II), Brookhaven National Lab (BNL). The chosen wavelength of the X-ray was 0.9Å and the sample-to-detector distance was 3.6 m. The sample was injected to a liquid sample holder with mica as the window materials. Five different positions were selected to reduce the experimental errors, and an exposure time of 1 s was set for each position. A polydispersed parallelepiped model was used to fit the scattering curve and extract the cross-sectional dimensions and distributions according to **Fig S1**.

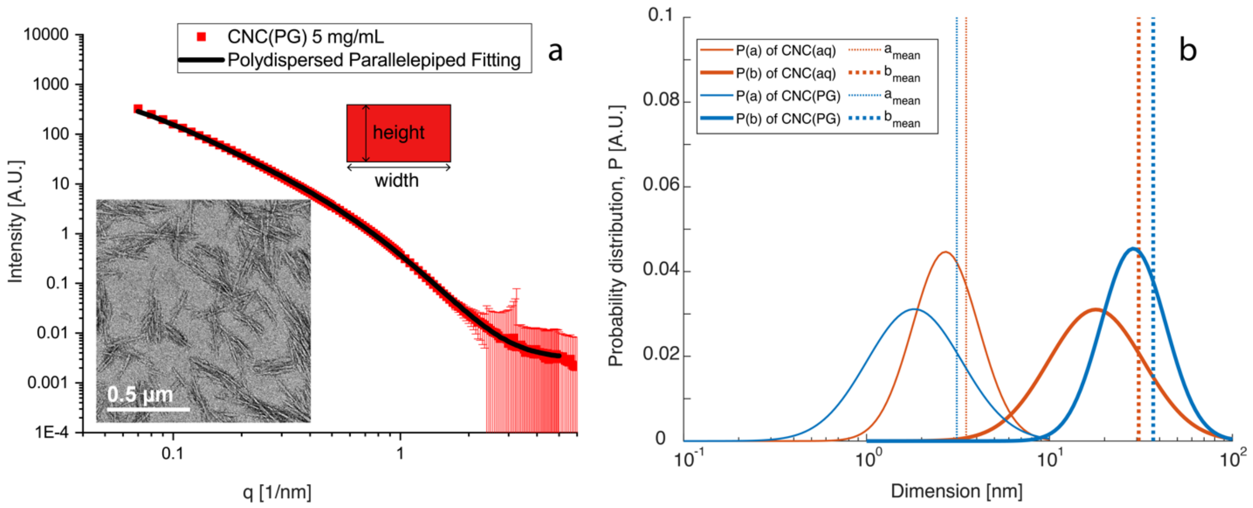

Figure S1. Analysis of CNC cross-sections through SAXS; (a) the scattering curve of CNC(PG) at 5 mg/mL and the polydisperse parallelepiped fitting; the inset figure is the TEM image of individual CNC particles; (b) the mean size and the size distribution of cross-sectional dimensions of CNCs in water and PG; CNCs exhibited the mean cross-sectional dimensions of 3.5×30.7 nm^2 (blue dashed line) in water and 3.1×37.0 nm^2 (orange dashed line) in PG.

Post-Processing of Scanning-SAXS Experiments

The scattering invariant was calculated by following:[1]

$$Q^* = \int_{0.06nm^{-1}}^{1.5nm^{-1}} I(q) \times q^2 dq$$

where $I(q)$ is the scattering intensity and q is the scattering vector. The normalized scattering intensity is obtained by $I^* = I/Q^*$. The anisotropic factor (AF) was calculated as:[2]

$$q_x q_x = \frac{\iint I \times q^2 \times \cos\chi \times \cos\chi \, d\chi dq}{\iint I \times q^2 d\chi dq}$$

$$q_x q_y = \frac{\iint I \times q^2 \times \cos\chi \times \sin\chi \, d\chi dq}{\iint I \times q^2 d\chi dq}$$

$$q_y q_y = \frac{\iint I \times q^2 \times \sin\chi \times \sin\chi \, d\chi dq}{\iint I \times q^2 d\chi dq}$$

$$AF = \sqrt{(q_x q_x - q_y q_y)^2 + 4 \times (q_x q_y)}$$

where χ is the azimuthal angle of the detector plane in polar coordinate. The q range is from 0.25 nm^{-1} to 0.45 nm^{-1} and the χ range is from $-\pi/2$ to $\pi/2$. The idea of the AF is that it does not need an aligned reference direction and the degree of the anisotropy could be directly obtained from the original 2D scattering pattern.

The structure parameter ϒ was calculated by the following steps:

1. The normalized form factor $P^*(q)$ is defined as the scattering of dilute CNC(aq) at 0.4 wt% from the work of Rosén *et al.*[1] normalized with its scattering invariant Q^*.

2. The structure factor $S^*(q)$ was obtained by dividing $I^*(q)$ with $P^*(q)$:

$$S^*(q) = \frac{I^*(q)}{P^*(q)}$$

$S^*(q)$ was to illustrate the deviation of CNC structures from a dilute disordered system.

3. The structure parameter ϒ was calculated as follow:

$$\Upsilon = \int_{0.06nm^{-1}}^{0.4nm^{-1}} \left| \frac{dS^*(q)}{dq} \right| dq$$

where the ϒ is used to quantify the degree of deviation of $S^*(q)$ from a dilute system.

The structure size d was obtained the same way as described by Rosén *et. al.*[1] $S^*(q)$ was firstly smoothed using a spline function and the q position where $S^*(q)$ reached the max value was located within the q range of from 0.06 nm^{-1} to 0.25 nm^{-1} (denoted as q_d). The value of d was obtained by:

$$d = \frac{2\pi}{q_d}$$

Spatially Resolved Quantities from Scanning-SAXS at Other Flow Rates

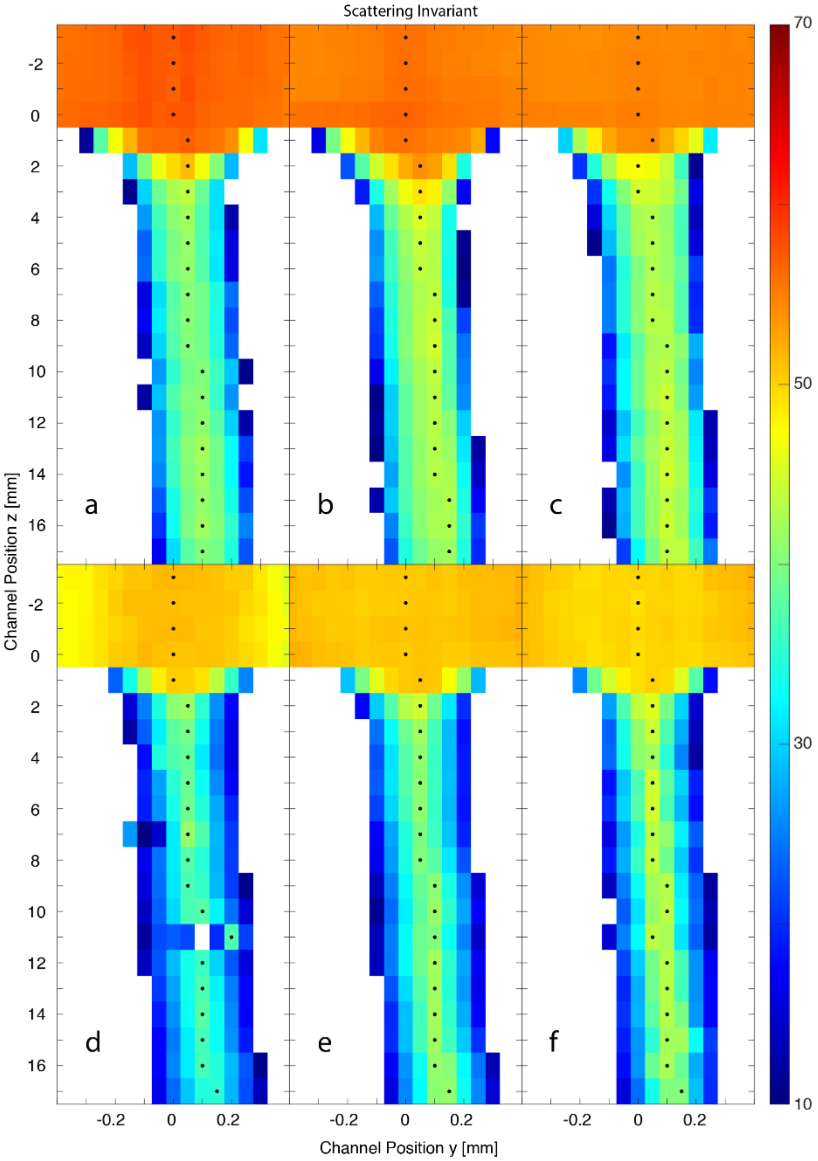

Figure S2. The scattering invariant Q^* of CNC(aq) at (a) 10 mL/h, (b) 20 mL/h and, (c) 50 mL/h, and CNC(PG) at (d) 2 mL/h, (e) 4 mL/h, (f) 10 mL/h.

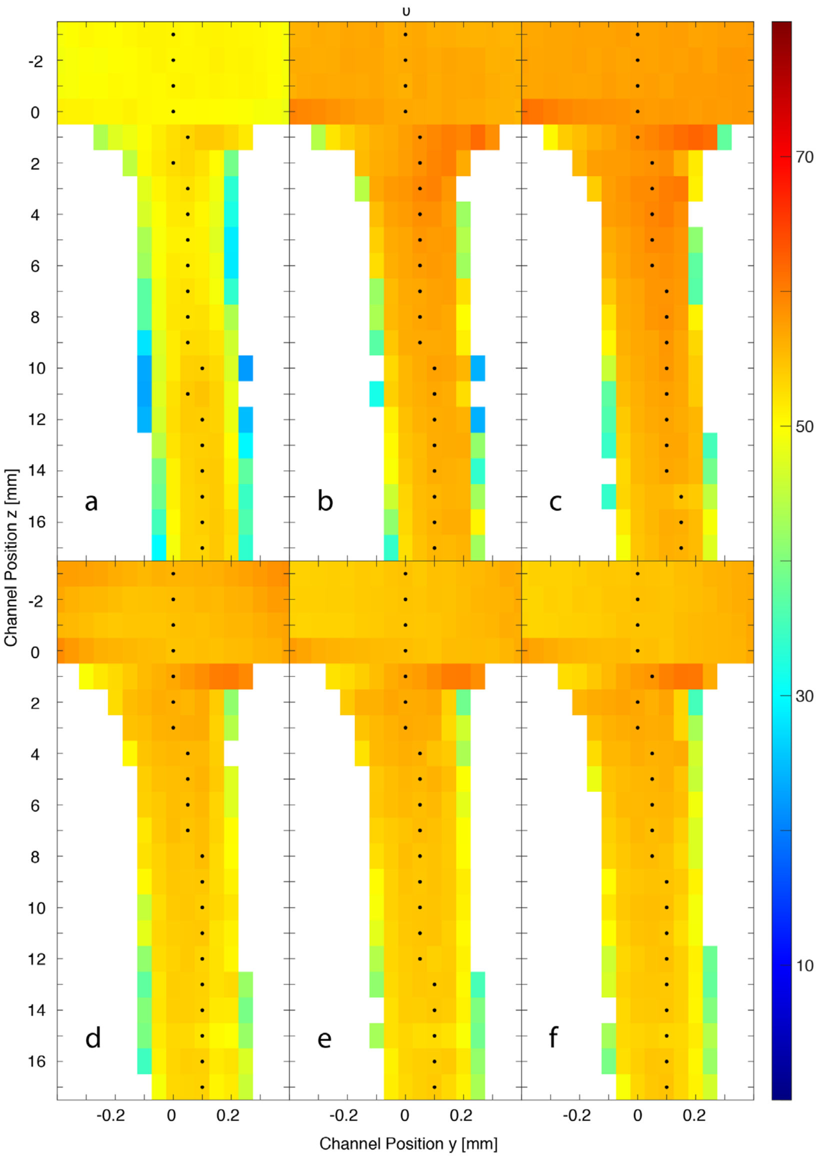

Figure S3. The structure parameter ϒ of CNC(aq) at (a) 5 mL/h, (b) 10 mL/h, (c) 20 mL/h, (d) 30 mL/h, (e) 40 mL/h, and (f) 50 mL/h; the reason why CNCs at 5 mL/h shows smaller ϒ, seems to be related to the low alignment and that artefacts during the azimuthal averaging of $I(q)$ leads to difficulties comparing the values between isotropic and aligned systems, since the value correlates strongly to alignment.

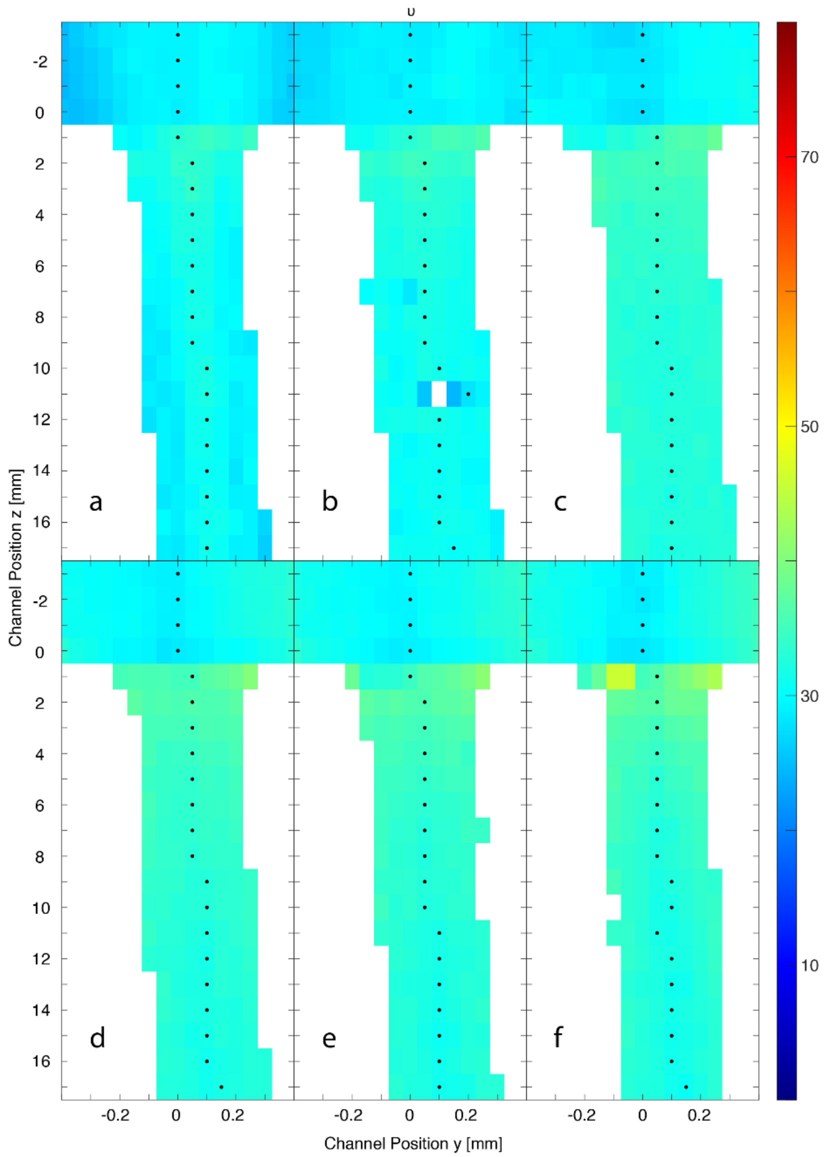

Figure S4. The structure parameter Υ of CNC(PG) at (a) 1 mL/h, (b) 2 mL/h, (c) 3 mL/h, (d) 4 mL/h, (e) 5 mL/h, and (f) 10 mL/h.

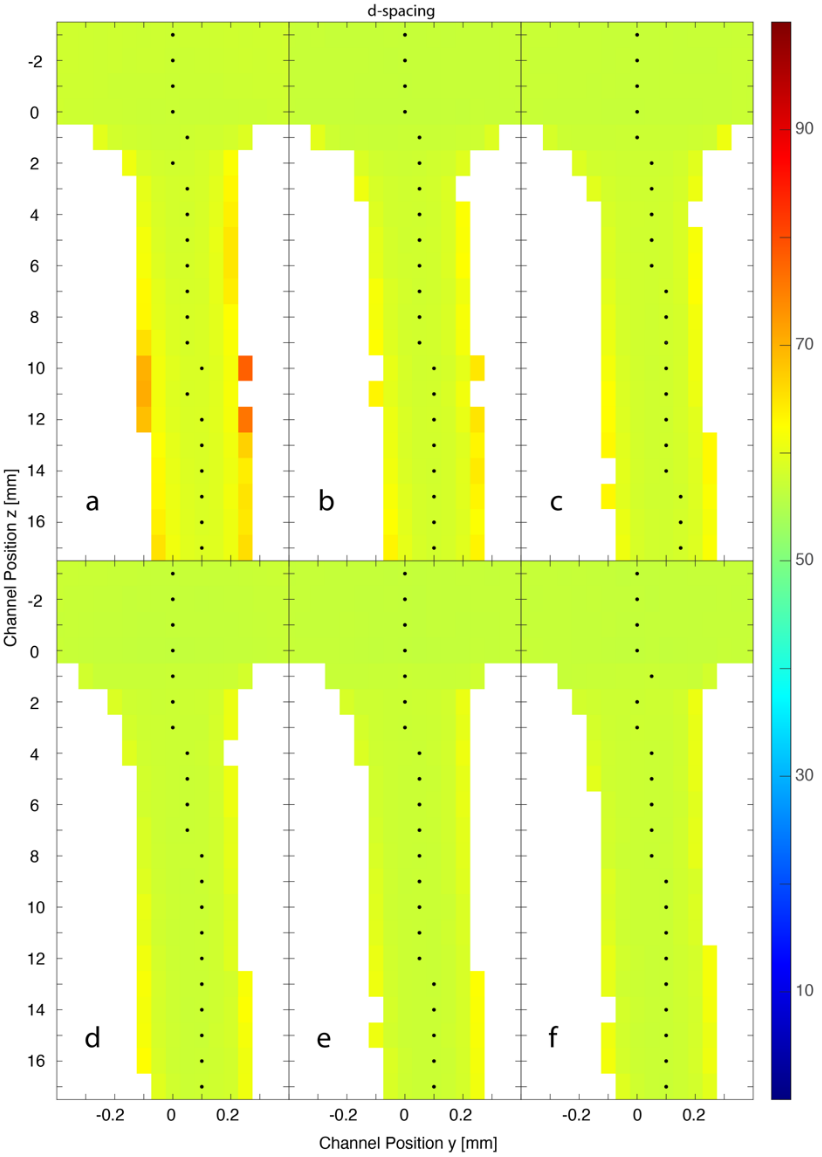

Figure S5. The d spacing of CNC(aq) in tactoids at (a) 5 mL/h, (b) 10 mL/h, (c) 20 mL/h, (d) 30 mL/h, (e) 40 mL/h, and (f) 50 mL/h.

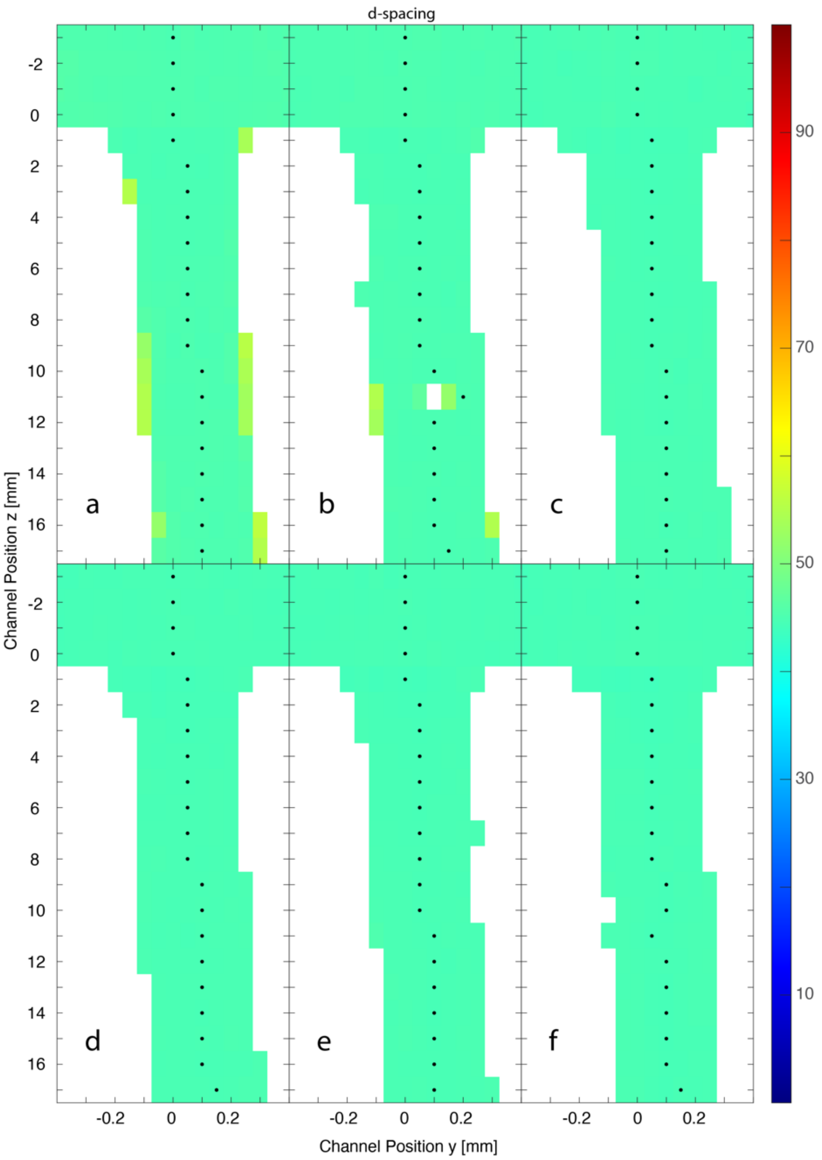

Figure S6. The spacing d of CNC(PG) in tactoids (a) 1 mL/h, (b) 2 mL/h, (c) 3 mL/h, (d) 4 mL/h, (e) 5 mL/h, and (f) 10 mL/h.

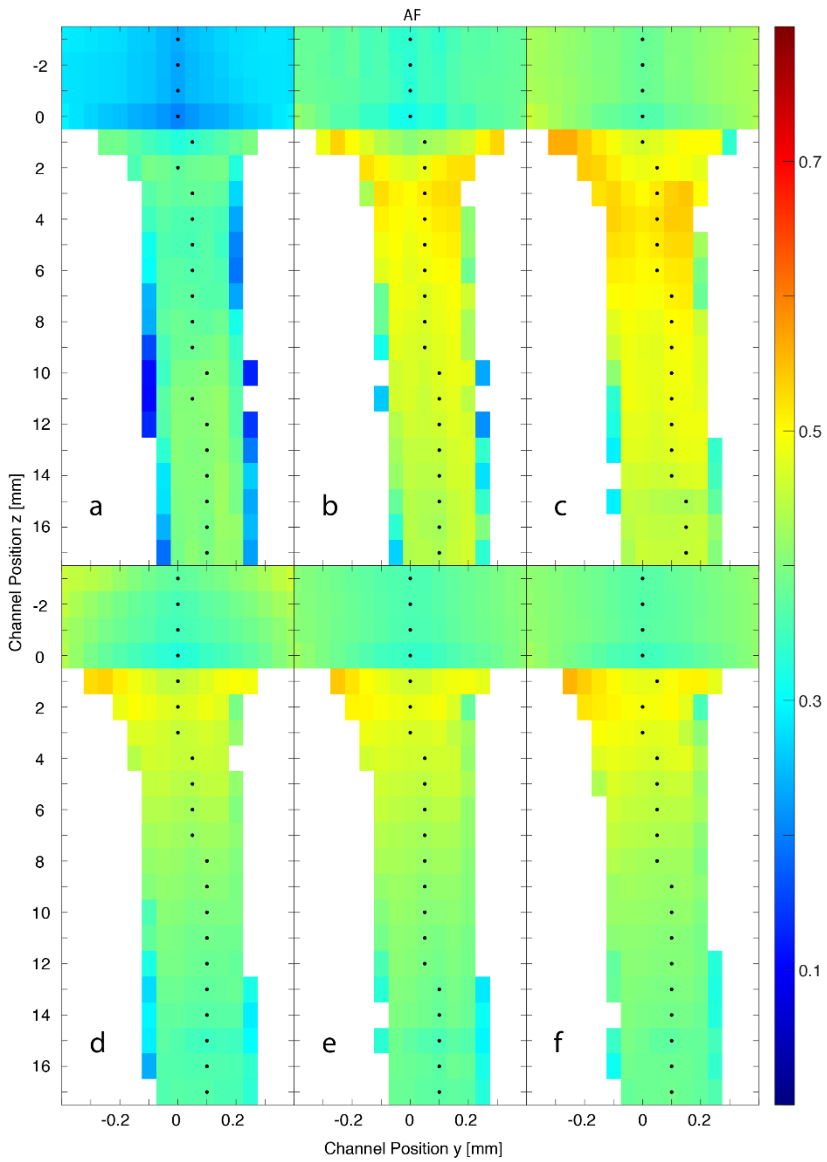

Figure S7. The anisotropic factor *AF* of CNC(aq) at (a) 5 mL/h, (b) 10 mL/h, (c) 20 mL/h, (d) 30 mL/h, (e) 40 mL/h, and (f) 50 mL/h.

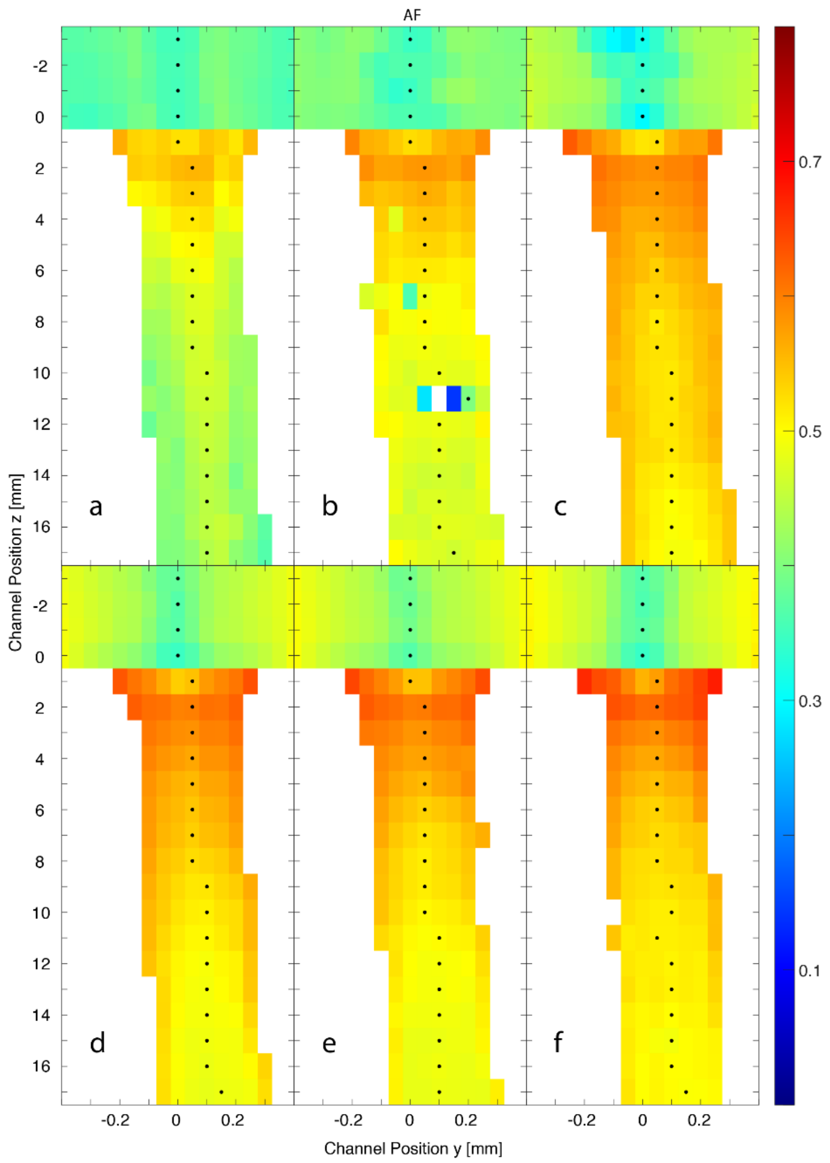

Figure S8. The anisotropic factor *AF* of CNC(PG) at (a) 1 mL/h, (b) 2 mL/h, (c) 3 mL/h, (d) 4 mL/h, (e) 5 mL/h, and (f) 10 mL/h.

Rheo-optical Flow-Stop

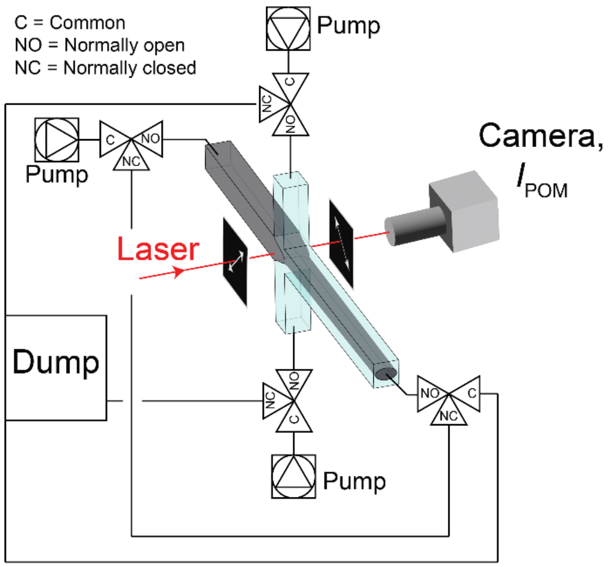

Figure S9. Illustration of the rheo-optical flow-stop experiment by Rosén *et al*;[3] the instrument was set such that 1 mm corresponds to 50 pixels on the camera.

The experimental setup of the rheo-optical experiment is illustrated in **Fig S9**. In this setup, the light generated by the laser module can be decomposed into components polarized perpendicular and parallel to the flow direction (z-direction). As the aligned CNCs become birefringent,[4] *i.e.*, $n_{\parallel} \neq n_{\perp}$, the difference between 2 refractive indices ($\Delta n = n_{\parallel} - n_{\perp}$) will cause a phase shift $\Delta\varphi$ of two components of the light passing through, which can be quantified by placing two linear polarizing filters on each side of the flow cell oriented at $+45°$ and $-45°$ to the z-direction. As a result, the transmitted light intensity (I) can be related with the Δn through an approximation:[5]

$$I = I_0 \sin^2\left(\frac{2\pi d \Delta n}{\lambda}\right)$$

where d is the distance that the light is travelled in the CNC (d was used as the structure size earlier, please change one symbol), λ is the wavelength of the light and I_0 is the maximum transmitted intensity, which can be obtained at a phase shift of 90 degrees according to the procedure in the main manuscript. The birefringence is directly proportional to the order parameter $S_\phi = \frac{1}{2}\langle 3\cos\phi - 1\rangle \propto \Delta n$, with ϕ being the angle between CNC major axis and flow direction, and the brackets denoting an ensemble average. The decay of Δn after stop will thus reflect the Brownian rotary diffusion of CNCs, which in an ideal dilute case of monodisperse rods decays as $\Delta n \propto S_\Phi \propto \exp(-6D_r t)$, with D_r being the rotary diffusion coefficient.

In the present study, the decay is not fully exponential, and some degree of polydisperse dynamics must be considered. The cumulant expansion tool is to re-write a sum of exponential decay functions as a power series expansion according to:

$$\log(\Delta n) = \log(\Delta n_0) - 6D_{mean}t + 2 \cdot PDI \cdot (6D_{mean})^2 t^2$$

where t is the time after the flow was stopped, Δn_0 is the birefringence level when flow was just stopped (a relaxing time of 50 ms is considered to avoid artefacts during the shutting of the valves), D_{mean} is the mean rotary diffusion coefficient and PDI is the polydispersity index.

The strong birefringence of the material caused a value of $\frac{2\pi d\Delta n}{\lambda} > 90°$, leading to an oscillating intensity during the decay. This phenomenon is illustrated in a supplementary video below. However, by assuming a constant decay of Δn, the conversion from intensity to birefringence could be easily done through identifications of shifts of $90°, 180°, 270°$ etc. The procedure is illustrated in **Fig S10**.

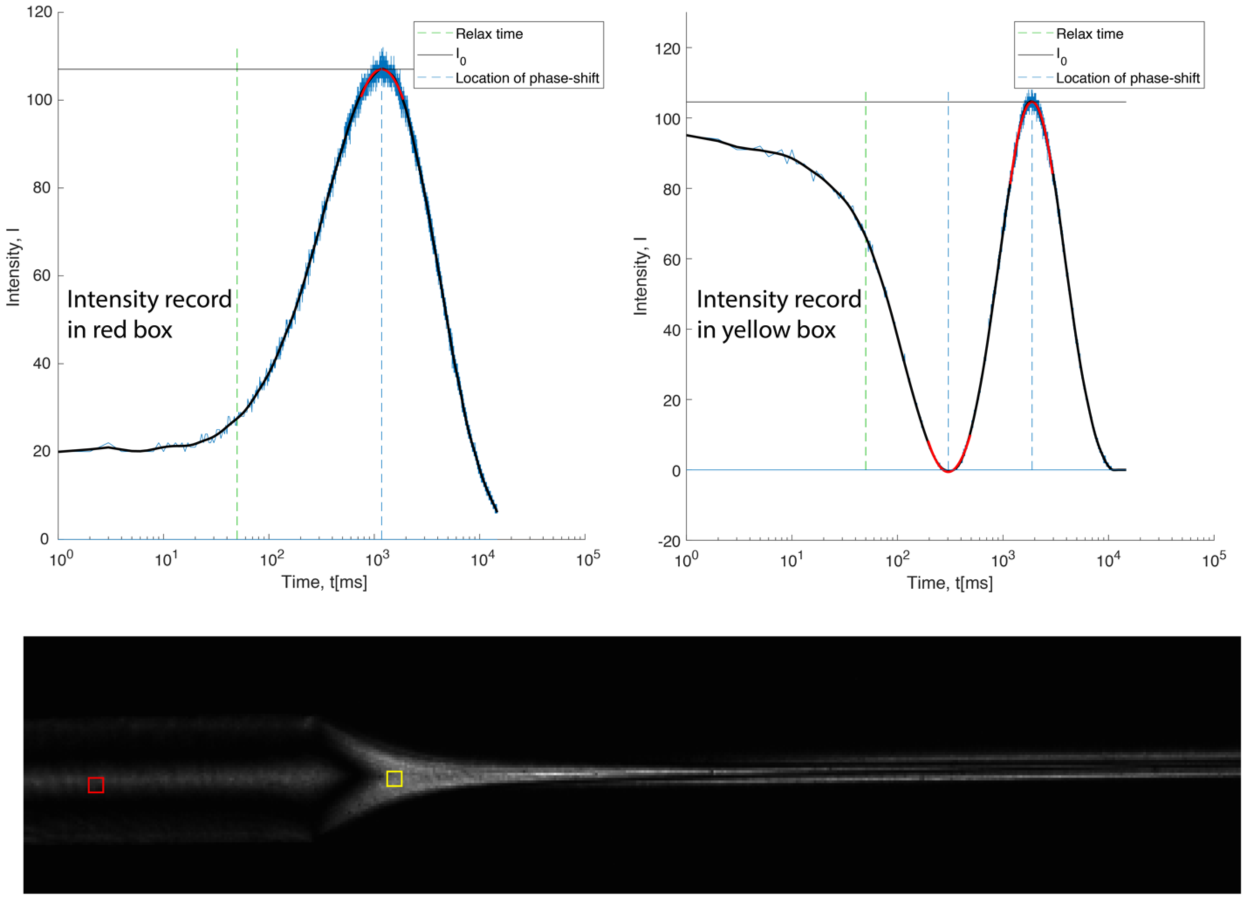

Figure S10. The extracted intensity (the upper two figures, blue line is extracted intensities, the black line is the spline fitting of the intensities and the red curve indicates the small phase shift) at center of the upper stream (red box) and focusing region (yellow box) of the lower image.

Polydispersity Index, PDI

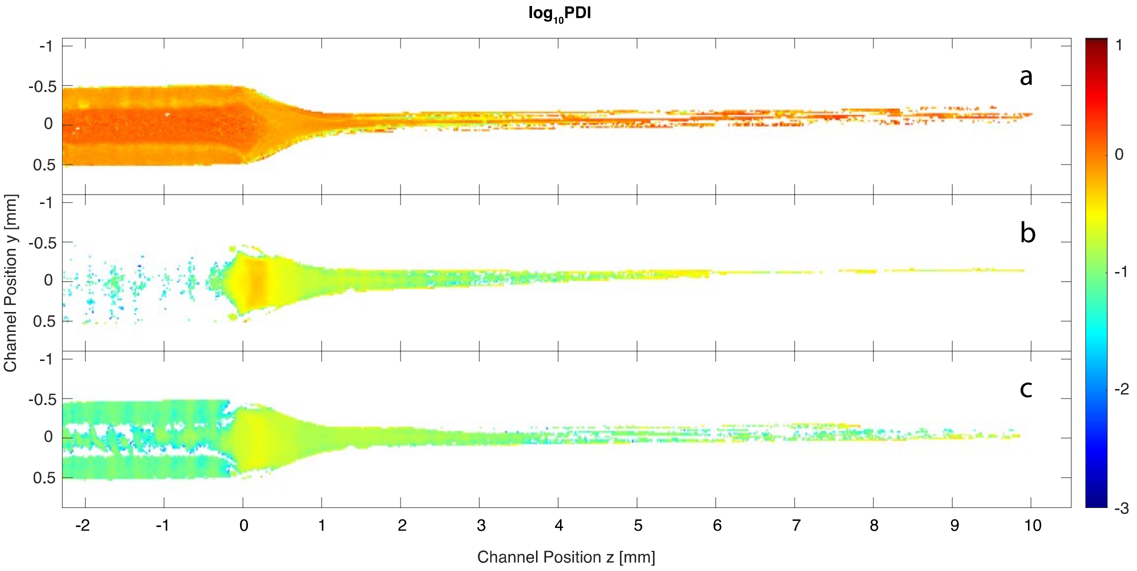

Figure S11. The PDI maps of (a) CNC(PG) at 10 mL/h, (b) CNC(aq) at 10 mL/h, and (c) CNC(aq) at 50 mL/h.

An example of spatially resolved PDI-values are illustrated in **Fig S11**. Generally, larger PDI means a wider size distribution. If comparing the PDI maps of CNC(aq) at 50 mL/h and 10 mL/h prior to the focusing region, CNC(aq) at 50 mL/h gave out more pixels with valid PDI values. When combining with the D_{mean} values of CNC(aq) at corresponding flow rates, D_{mean} at 50 mL/h became larger than 10 mL/h, with a broader distribution of dynamic time scales. And because D_{mean} is inversely related to the structural size, it can be concluded that high flow rates could align both small and large tactoids, which contributed to the broader distribution of D_{mean}. While at the focusing region, CNC(aq) at 50 mL/h gave a smaller PDI and a larger D_{mean}. As high flow rates could break up the CNC mesoscopic structure (the hypothesis from the main manuscript), the major population of aligned CNCs was in small size. Here the small size could refer to both individual CNC rod and smaller CNC tactoidal structure. And the fact of the small size contributed

to both a small PDI and a large D_{mean} at the focusing region and flow rate of 50 mL/h. While for CNC(aq) at 10 mL/h, the mesoscopic structures were present, and they are more easily jammed because of the deceleration accompanied with the compression (**Fig 1**(a) in main manuscript, region II). Moreover, as smaller tactoids coalesce into bigger ones will also lead to a wider distribution of the D_{mean}. As a conclusion, larger structures resulted in a smaller D_{mean} at focusing region and flow rate of 10 mL/h, but the possibilities of small tactoid fusion contributed to a large PDI.

For CNC(PG) at 10 mL/h, the system behaved very differently. The PDI of CNC(PG) was much larger than CNC(aq) at the same flow rate, meaning a much wider D_{mean} distribution of the aligned materials. This indicated that a wider size distribution of CNC tactoids was aligned owing to low solvent viscosity. And the PDI did not show obvious change with channel positions. At the focusing region, PDI of CNC(PG) exhibits a slight decrease, which may be caused by the flow deceleration accompanied with the compression.

Theoretical Estimation of $D_{r,0}$

The theoretical rotary diffusion coefficient of dilute monodisperse spheroids can be obtained through:[6]

$$D_{r,0} = \frac{3k_bT(2\ln r_p - 1)}{2\pi\eta L^3}$$

where $D_{r,0}$ is the rotary diffusion coefficient, k_b is the Boltzmann constant, T is the temperature in Kelvin, r_p is the aspect ratio, η is the solvent viscosity and L is the length of the CNCs. From our previous work,[7] we know that the dilute CNCs behave like Brownian spheroids of dimensions

$L = 150$ nm and $r_p = 15$. Using $T = 293$ K, $\eta_{PG} = 0.042$ Pa s and $\eta_{water} = 0.00089$ Pa s, we estimate that $D_{r,0} = 60.2$ rad^2/s in PG and $D_{r,0} = 2840$ rad^2/s in water.

Computational Fluid Dynamics (CFD)

To illustrate the shear rate distribution in the channel, CFD was conducted on COMSOL Multiphysics software. The geometry of the channel is 5 mm long and 1 mm wide, with a square cross-section shape. A laminar flow was assumed to perform the simulation.

The incompressible steady-state laminar flow in a square channel could be predicted by Navier–Stokes equation in the specific form:

$$\rho(\boldsymbol{u} \cdot \nabla)\boldsymbol{u} = \nabla \cdot [-p\mathbf{I} + \mathbf{K}]$$

where $\boldsymbol{u}$ is the flow velocity, $\mathbf{K}$ is the stress tensor, p is the pressure and $\mathbf{I}$ is the identity matrix. For the incompressible fluid, we have the following equation:

$$\rho\nabla \cdot \boldsymbol{u} = 0$$

$\mathbf{K}$ could be obtained by:

$$\mathbf{K} = \eta_{app}(\dot{\gamma})(\nabla\boldsymbol{u} + (\nabla\boldsymbol{u})^{\mathrm{T}})$$

where $\eta_{app}(\dot{\gamma})$ is the apparent dynamic viscosity as function of shear rate $\dot{\gamma}$ of the suspension obtained by the rheological experiments, (see **Fig S14**). The resulting shear rate distribution is shown in **Fig S12** below.

The estimated shear rate at a certain projected channel height was taken as the value from the midplane in the channel, where the system is observed in vorticity direction and contributes more to the signal due to higher projected alignment.[8]

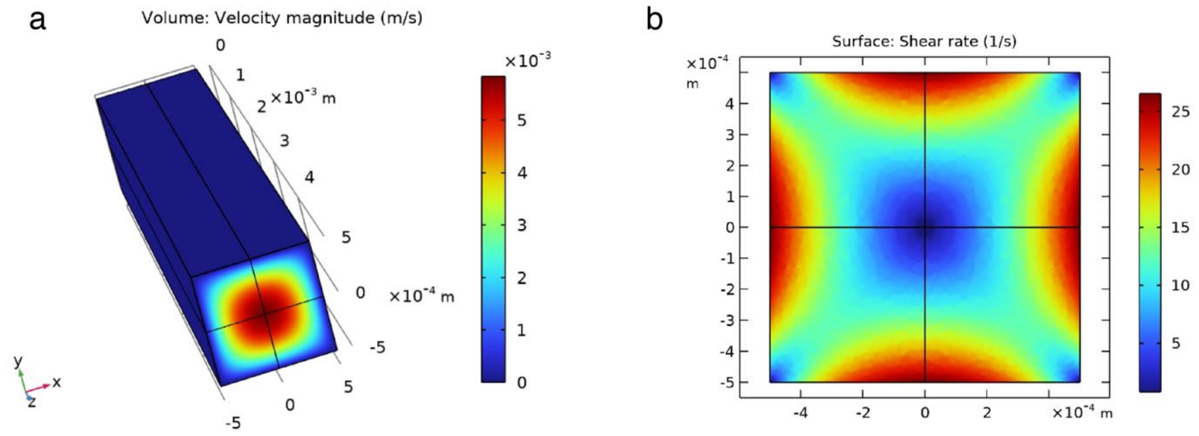

Figure. S12 Results from CFD simulations; (a) the velocity distribution in the channel for CNC(aq) at 10 mL/h, and (b) the shear rate distribution on X-Y plane of CNC(aq) at 10 mL/h.

Analyzing Values of D_{mean} at Different Flow Rates

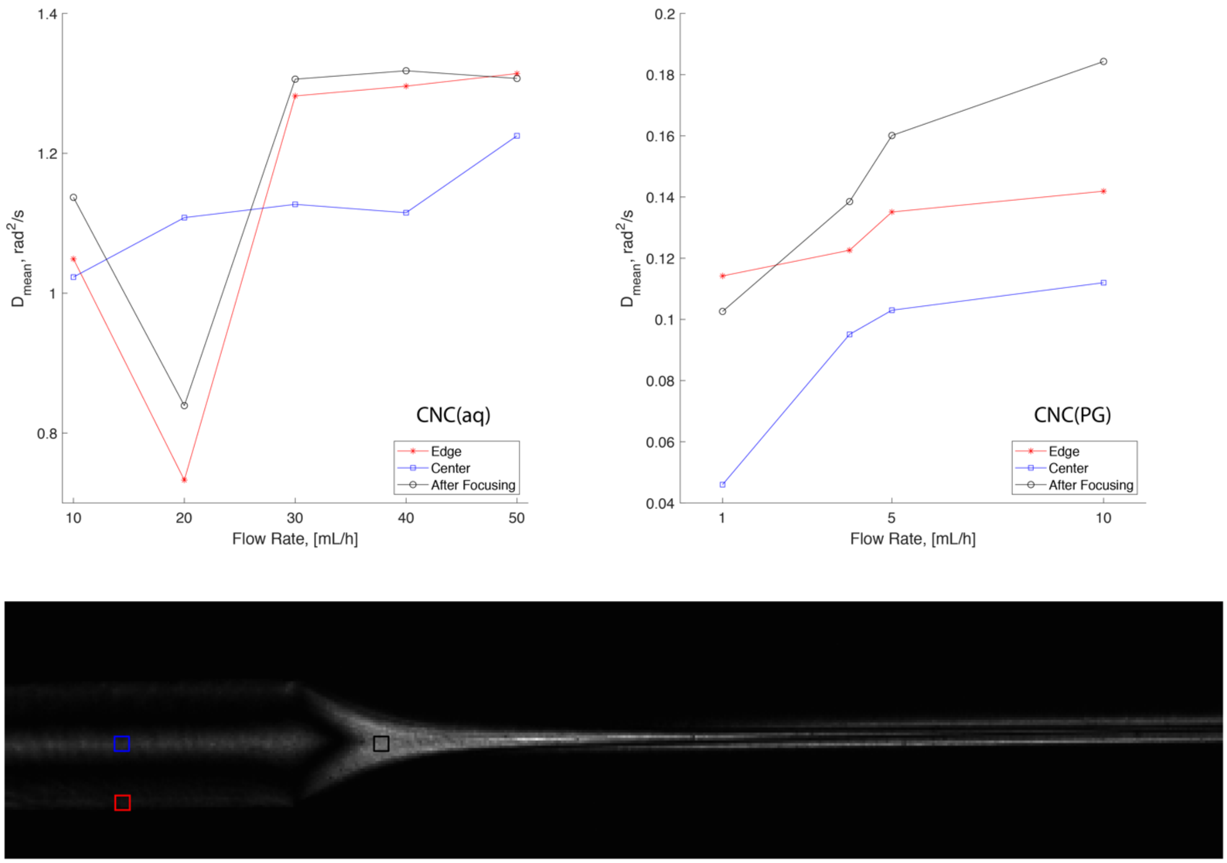

Figure S13. The plot of D_{mean} at different positions versus the flow rates for both CNC(aq) (upper left figure) and CNC(PG) (upper right figure); the positions are located: at the wall prior to the focusing (red box), at the center prior to the focusing (blue box), and at the center right after the focusing (black box); note that the value of D_{mean} in the aqueous system seems to be limited at 1.3 rad^2/s.

Rheological Measurements

The viscosity of the CNC dispersed in aqueous (CNC(aq)) and propylene glycol (CNC(PG)) was obtained by a stress-controlled rheometer (Physica MCR-301, Anton Paar). The concentric cylinder geometry with the operating gap of 1.12 mm was used in this study. The steady shearing experiment was carried out in a sequence of shear rate from 1 s^{-1} to 1000 s^{-1} with 10 different shear rates per decade and 10 seconds at each shear rate. The result is illustrated in **Fig S14**.

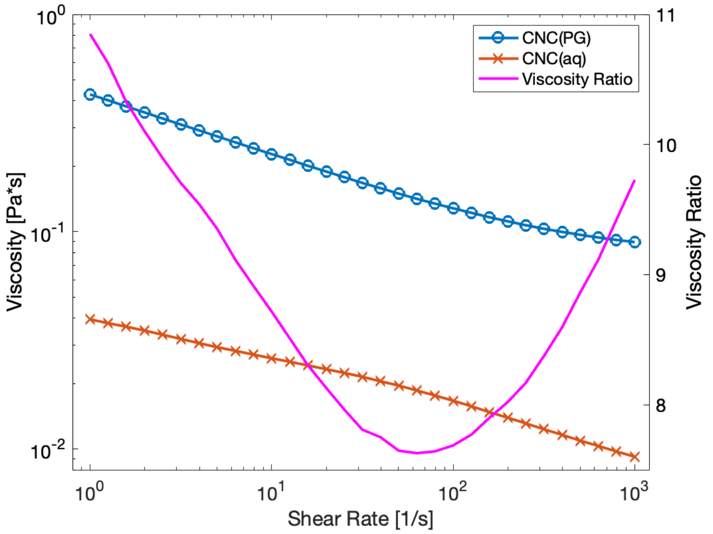

Figure S14. The steady viscosity profile of CNC(aq) and CNC(PG) in log scale at concentration of 36 mg/mL; the viscosity ratio of CNC(PG) over CNC(aq) is shown as the magenta curve.

A power law fitting was used to obtain the relationship between shear rate and the viscosity for both CNC(aq) and CNC(PG). The equation is as shown:

$$\eta_{app}(\dot{\gamma}) = m\dot{\gamma}^{n-1}$$

where m and n are fitting parameters and $\dot{\gamma}$ is the shear rate. The fitting results in relationships of $\eta = 0.4253 \cdot \dot{\gamma}^{-0.2681}$ for CNC(aq) and $\eta = 0.0396 \cdot \dot{\gamma}^{-0.1823}$ for CNC(PG). The fitting equations are valid within the range of 0 - 150 s^{-1}, which perfectly covers the achievable shear rates obtained from CFD.

References:

1. Rosén, T.; Wang, R.; He, H.; Zhan, C.; Chodankar, S.; Hsiao, B. S., Shear-free mixing to achieve accurate temporospatial nanoscale kinetics through scanning-SAXS: ion-induced phase transition of dispersed cellulose nanocrystals. *Lab Chip.* **2021,** *21* (6), 1084-1095.
2. Pujari, S.; Rahatekar, S.; Gilman, J. W.; Koziol, K. K.; Windle, A. H.; Burghardt, W. R., Shear-induced anisotropy of concentrated multiwalled carbon nanotube suspensions using x-ray scattering. *J. Rheol.* **2011,** *55* (5), 1033-1058.
3. Rosén, T.; Mittal, N.; Roth, S. V.; Zhang, P.; Lundell, F.; Söderberg, L. D., Flow fields control nanostructural organization in semiflexible networks. *Soft Matter* **2020,** *16* (23), 5439-5449.
4. Calabrese, V.; Haward, S. J.; Shen, A. Q., Effects of shearing and extensional flows on the alignment of colloidal rods. *Macromolecules* **2021,** *54* (9), 4176-4185.
5. Håkansson, K. M., Online determination of anisotropy during cellulose nanofibril assembly in a flow focusing device. *RSC Adv.* **2015,** *5* (24), 18601-18608.
6. Doi, M.; Edwards, S. F., *The theory of polymer dynamics*. oxford university press: 1988; Vol. 73.
7. Rosén, T.; Wang, R.; Zhan, C.; He, H.; Chodankar, S.; Hsiao, B. S., Cellulose nanofibrils and nanocrystals in confined flow: Single-particle dynamics to collective alignment revealed

through scanning small-angle x-ray scattering and numerical simulations. *Phys. Rev. E* **2020,** *101* (3), 032610.

8. Rosén, T.; Hsiao, B. S.; Söderberg, L. D., Elucidating the Opportunities and Challenges for Nanocellulose Spinning. *Adv. Mater.* **2021,** *33* (28), 2001238.
